# Porcine ZBED6 regulates growth of skeletal muscle and internal organs via multiple targets

**DOI:** 10.1371/journal.pgen.1009862

**Published:** 2021-10-28

**Authors:** Dandan Wang, Dengke Pan, Baocai Xie, Shengnan Wang, Xiangyang Xing, Xuexue Liu, Yuehui Ma, Leif Andersson, Jiangwei Wu, Lin Jiang

**Affiliations:** 1 Laboratory of Animal (Poultry) Genetics Breeding and Reproduction, Ministry of Agriculture, Institute of Animal Sciences, Chinese Academy of Agricultural Sciences (CAAS), Beijing, China; 2 National Germplasm Center of Domestic Animal Resources, Ministry of Technology, Institute of Animal Sciences, Chinese Academy of Agricultural Sciences (CAAS), Beijing, China; 3 Clinical Immunology Translational Medicine Key Laboratory of Sichuan Province, Sichuan Academy of Medical Sciences & Sichuan Provincial People’s Hospital, Chengdu, China; 4 Key Laboratory of Animal Genetics, Breeding and Reproduction of Shaanxi Province, College of Animal Science and Technology, Northwest A&F University, Yangling, Shaanxi, China; 5 Chengdu Clonorgan Biotechnology Co. LTD, Chengdu, China; 6 Science for Life Laboratory, Department of Medical Biochemistry and Microbiology, Uppsala University, Uppsala, Sweden; 7 Department of Animal Breeding and Genetics, Swedish University of Agricultural Sciences, Uppsala, Sweden; 8 Department of Veterinary Integrative Biosciences, Texas A&M University, College Station, Texas, United States of America; University of Bern, SWITZERLAND

## Abstract

ZBED6 (zinc finger BED domain containing protein 6) is a transcription factor unique to placental mammals and its interaction with the *IGF2* (insulin-like growth factor 2) locus plays a prominent role in the regulation of postnatal skeletal muscle growth. Here, we generated lean Bama miniature pigs by generating *ZBED6*-knockout (*ZBED6*^*−/−*^) and investigated the mechanism underlying *ZBED6* in growth of muscle and internal organs of placental mammals. *ZBED6*^*−/−*^ pigs show markedly higher lean mass, lean mass rate, larger muscle fiber area and heavier internal organs (heart and liver) than wild-type (WT) pigs. The striking phenotypic changes of *ZBED6*^*-/-*^ pigs coincided with remarkable upregulation of IGF2 mRNA and protein expression across three tissues (gastrocnemius muscle, *longissimus dorsi*, heart). Despite a significant increase in liver weight, *ZBED6*^*-/-*^ pigs show comparable levels of *IGF2* expression to those of WT controls. A mechanistic study revealed that elevated methylation in the liver abrogates ZBED6 binding at the *IGF2* locus, explaining the unaltered hepatic *IGF2* expression in *ZBED6*^*-/-*^ pigs. These results indicate that a ZBED6-*IGF2*-independent regulatory pathway exists in the liver. Transcriptome analysis and ChIP-PCR revealed new ZBED6 target genes other than *IGF2*, including cyclin dependent kinase inhibitor 1A (*CDKN1A*) and tsukushi, small leucine rich proteoglycan (*TSKU*), that regulates growth of muscle and liver, respectively.

## Author summary

The lean meat rate is an important economic trait for the swine industry and it is determined by muscle growth and development. A single base change in intron 3 of the insulin-like growth factor 2 (*IGF2*) gene increases meat production in pigs by disrupting a binding site for zinc finger BED domain containing protein 6 (ZBED6). Chinese indigenous pig breeds carrying the homozygous *IGF2* wild-type allele produce low lean meat. We thus generate a lean pig model in Chinese Bama pig by knocking out *ZBED6*. In this model, we demonstrate that *ZBED6* KO increases muscle and internal organ growth through ZBED6-*IGF2* axis and other target genes. These results not only open new strategies for lean meat breeding in Chinese indigenous pigs, but also provide new insights to the global function of ZBED6 in organ growth and development.

## Introduction

Modern commercial pigs have increased skeletal muscle mass and reduced backfat thickness due to the strong selection for lean meat production. The gene for insulin-like growth factor 2 (*IGF2*) underlies a paternally expressed quantitative trait locus in pigs and the causal mutation is a single nucleotide transition from G to A in intron 3 [1–3]. The mutant allele at this locus increases muscle mass, heart size and reduces fat deposition in pigs. The mutation, located in an evolutionarily conserved CpG island, abrogates a binding site for zinc finger BED domain containing protein 6 (ZBED6) and results in a 3-fold greater postnatal expression of *IGF2* mRNA in skeletal muscle [4]. The mutation has gone through a selective sweep due to strong selection for lean meat content and is fixed or close to fixation in the major breeds used for meat production worldwide such as Large White, Landrace and Hampshire [3]. In contrast, most indigenous Chinese pig breeds without intensive selection for growth and carcass traits are homozygous for the *IGF2* wild-type allele [5]. *ZBED6* has also been shown to regulate *IGF2* mRNA expression and insulin production in several human and murine cell lines [6–9]. A recent mouse study showed that *ZBED6* knockout (*ZBED6* KO) and *IGF2* knockin (*IGF2* KI) cause a very similar upregulated expression of *IGF2*, leading to enhanced growth of skeletal muscle and heart [10]. Furthermore, the same *IGF2* intron 3–3072 site knock-in improved meat production in pigs [11,12]. Chromatin immunoprecipitation (ChIP) sequencing using murine C2C12 cells indeed demonstrated that ZBED6 may regulate additional 1200 ZBED6 binding sites other than *IGF2*[4]. *ZBED6* regulates beta cell area and excess mitochondrial activation by controlling the cell cycle gene *PTTG1* and the mitochondrial regulator *PPAR-γ* related coactivator 1 protein (PRC) in mice, which occurred independently from *ZBED6* effects on *IGF2* gene expression [13]. However, the functional role of ZBED6 in pig besides its important role for regulating *IGF2* expression is still poorly characterized.

In the present study, to investigate the global function of *ZBED6* in pigs, we generated *ZBED6* knockout (*ZBED6*^*-/-*^) Bama miniature pigs using the CRISPR/Cas9 (clustered, regularly interspaced, short-palindromic repeats/CRISPR-associated protein 9) technique and obtained the F1-F4 generation by further crossing mutant pigs. We first measured carcass traits in the *ZBED6* knockout founder and F4 pigs to confirm the function of ZBED6 in regulating the growth of skeletal muscle and internal organs. We then performed transcriptome analysis in four tissues to identify *ZBED6* target genes other than *IGF2* that might explain the phenotypic effects in the *ZBED6* knockout pigs, such as increased liver and muscle growth.

## Results

### Generation of *ZBED6*^*-/-*^ pigs

**Fig 1:**
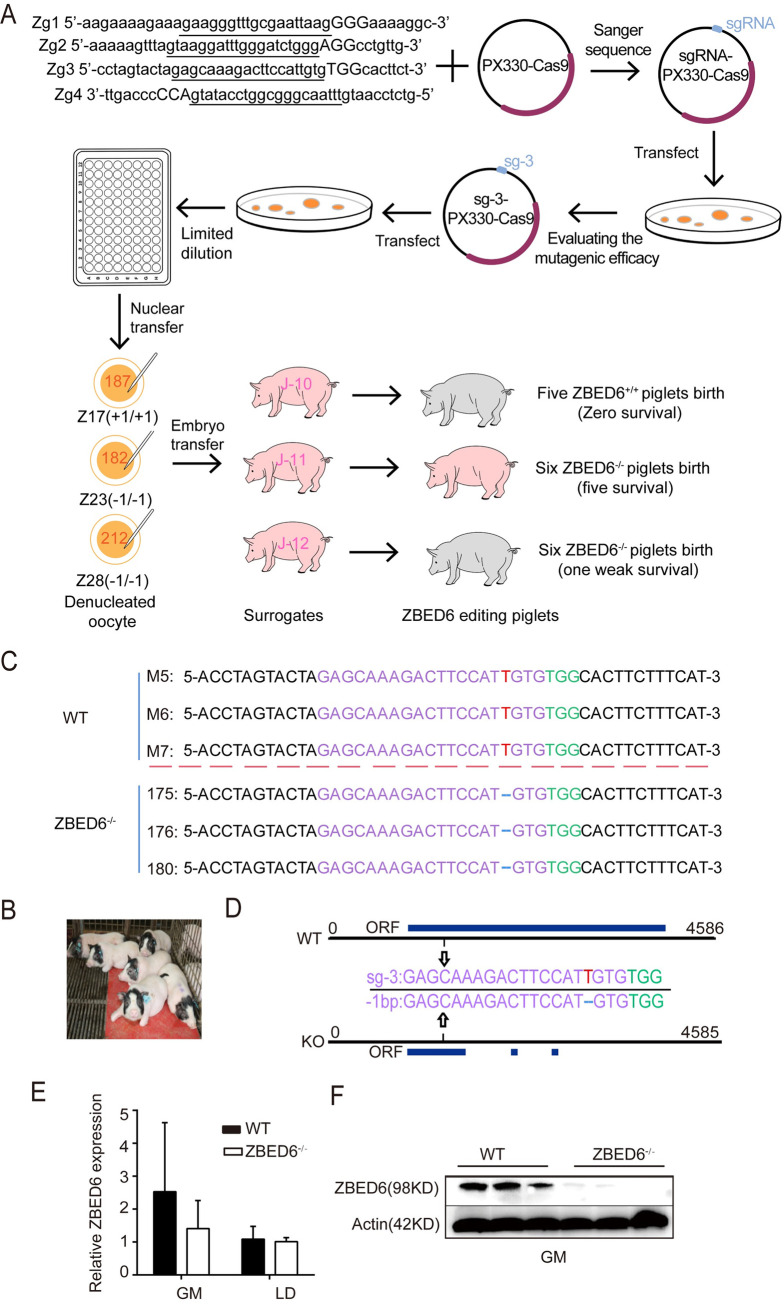
CRISPR/Cas9-mediated, nonhomologous end joining-independent integration efficiently produces *ZBED6*^-/-^ pigs. (n = 3) **(A)** Schematic overview of the production of *ZBED6* knockout pigs. Zg1, Zg2, Zg3 and Zg4 are four different sgRNAs, underline letters represent target sequences, capital letters represent the PAM sequence. The purple arrow and blue arrow represent the location of Cas9 and sgRNA, respectively. The *ZBED6* editing piglet in pink means the healthy piglets that were used as the test subjects in subsequent experiments. The *ZBED6* editing piglets in grey mean the piglets are weak or not survived. **(B)** *ZBED6* knockout 3-month-old pigs. **(C)** Genotyping of the *ZBED6* knockout pigs by polymerase chain reaction (PCR). SgRNA sequences are in purple type, PAM sequences are in cyan type, the light blue box represents the one-base pair deletion site (red T base).**(D)** Schematic representation of the knockout. The black represents *ZBED6* gene, the box blue bars represent the open reading frame (ORF), the black arrow represents the efficient sgRNA targeting site. **(E)** qPCR analysis (using *ZBED6* primer amplicons) of *ZBED6* mRNA of gastrocnemius muscle (GM) and *longissimus dorsi* (LD) tissues in *ZBED6*^-/-^ pigs. **(F)** Western blot analysis of ZBED6 in GM from *ZBED6*^-/-^ pigs. The results are the means ± SEMs. *p < 0.05; **p < 0.01, *** *p* < 0.001, Student’s t test.

A CRISPR/Cas9-mediated, nonhomologous end-joining-independent integration strategy was developed to knock out *ZBED6* in Bama miniature pigs (**Fig 1A**). Four sgRNAs Zg1, Zg2, Zg3 and Zg4, targeting at 1112-1132bp, 1175-1195bp, 1304-1327bp and 1350-1370bp of *ZBED6* respectively, were designed and individually transfected into porcine fetal fibroblasts (PFFs) by electroporation. Based on Sanger sequencing, the sgRNA3-pX330-Cas9 (Zg3) plasmid resulted in the best gene-targeting efficiency (~7.5%) and was thus transfected into PFFs to screen single-cell clones. Finally, 57 single-cell clones were obtained, and sequencing analysis showed that 39 (63.0%) positive cell clones included 11 monoallelic and 28 biallelic modifications of *ZBED6* (**S1 Table**). Three positive cell clones—Z17 (+1 bp/+1 bp), Z23 (-1 bp/-1 bp) and Z28 (-1 bp/-1 bp), in which Z23 and Z28 had the same deletion—were selected as donor cells for somatic cell nuclear transfer (**S1 Table**). Then, 187 (Z17), 212 (Z23) and 182 (Z28) reconstituted embryos were transferred into the oviducts of three surrogate sows, J10, J12 and J11, and sixteen piglets were given birth (J10: five, J12: six, J11: five). One weak (J12) and five healthy (J11) founder female piglets survived (**Fig 1B**). Five healthy founders were used as the test subjects in further experiments. Each cloned piglet was homozygous for a one-base pair deletion at position 1320-T of *ZBED6* (**Fig 1C**), which had been distinguished from WT pigs with high-resolution melting method [14]. This 1-bp deletion is a frameshift mutation, introducing the transcript was out of frame after codon 134 and a premature stop occurs at codon 224. This produces a short truncated protein (223 amino acids out of 981 amino acids in the full length protein = 22.7%) that did not maintain either of the two BED-DNA binding domains (**Fig 1D**). qPCR analysis showed that *ZBED6* transcripts had no significant expression changes (**Fig 1E**). Western blotting showed undetectable ZBED6 protein in the gastrocnemius muscle of *ZBED6*^-/-^ pigs (**Fig 1F**). To rule out the off-target effects, Sanger sequencing-based validation showed that none of the predicted off-target sequences exhibited mutations in *ZBED6*^-/-^ pigs (**S2 Table**). We also confirmed that the Bama pigs in this study are 100% fixed for the wild-type allele (G) at 3072 of *IGF2-*intron 3 (**S1A Fig**), which is important as the presence of the mutant allele would have a large effect on *IGF2* expression.

### *ZBED6*^-/-^ promotes skeletal muscle as well as internal organ growth

**Fig 2:**
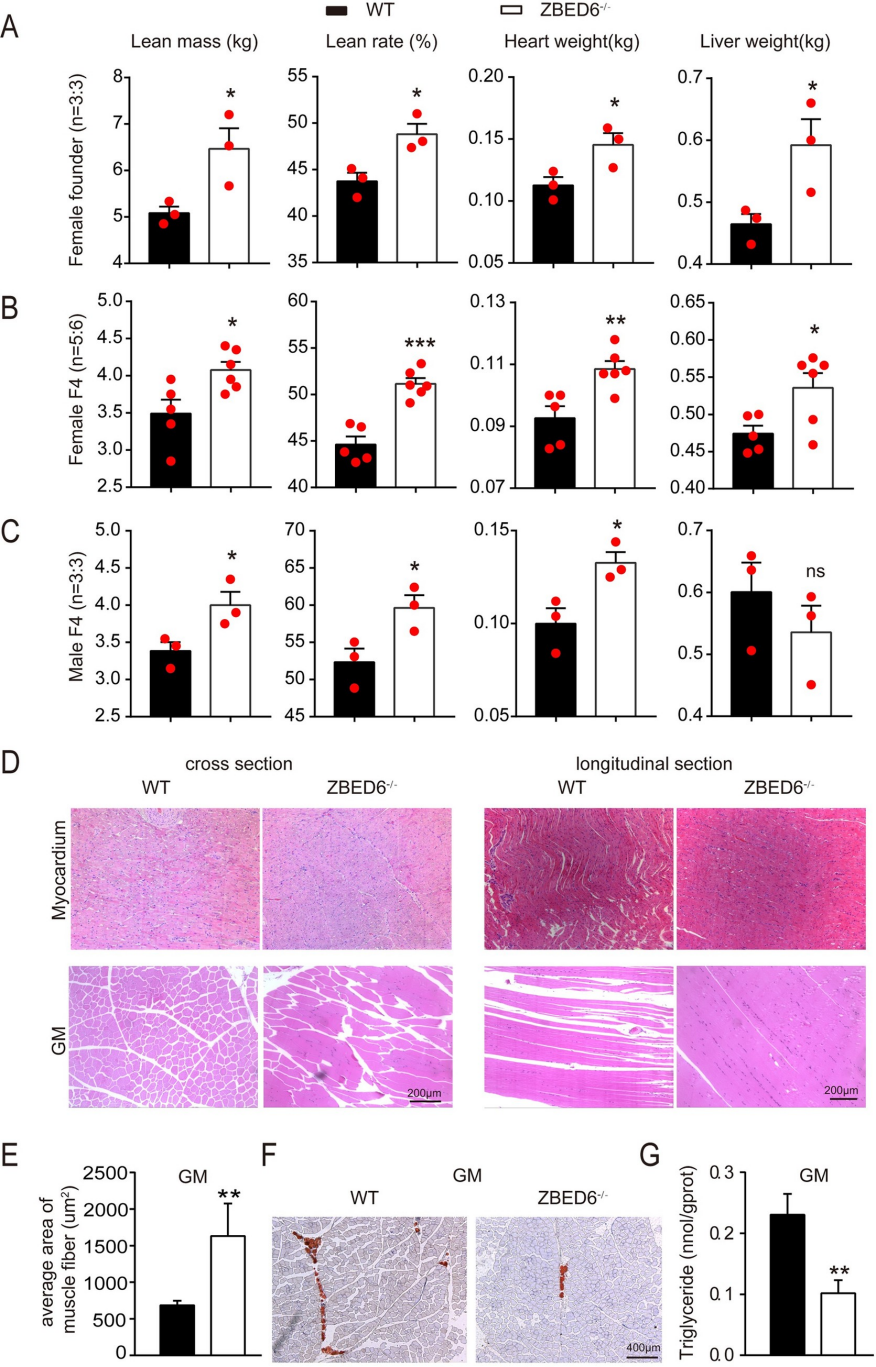
Phenotypes of slaughtered *ZBED6*^-/-^ founder and F4 pigs. **(A-C)** Dissected lean mass, lean rate, heart weight and liver weight of WT and *ZBED6*^*-/*-^ in female founders, female and male F4 pig littermates. Red points represent actual data of carcass traits. **(D)** H&E staining of the myocardium and gastrocnemius muscle (GM) of WT and *ZBED6*^-/-^ founder pigs. Bar, 200 μm. **(E)** Changes in GM fibre area in WT and *ZBED6*^-/-^ founder pigs. **(F)** Oil red O staining of GM in WT and *ZBED6*^-/-^ pigs. Bar, 400 μm. **(G)** Triglycerides of GM in WT and *ZBED6*^-/-^ pigs. The results are the means ± SEMs. *p < 0.05; **p < 0.01, *** *p* < 0.001, Student’s t test.

*ZBED6*^-/-^ pigs of founder and F4 showed similar body weight as the WT from birth to six months of age (**S1B Fig**). To further characterize *ZBED6*^-/-^ pigs, female founders (n = 3:3) at 8 months of age, female F4 (n = 5:6) and male F4 (n = 3:3) pigs at 5 months of age were slaughtered. The details of the carcass traits shown in **S3 Table** suggested the phenotype change of the founders is the same to that observed in F4 pigs. Greater lean mass (female founder: 27.28%, female F4: 16.76% and male F4: 18.24%), higher lean rate (female founder: 5.1%, female F4: 6.6% and male F4: 7.3%) and heavier hearts (female founder: 28.3%, female F4: 17.2% and male F4: 33%) were observed in all three groups of *ZBED6*^-/-^ pigs than in WT pigs (*p* < 0.05, **Fig 2A and 2C**). The percentage of lean rate increase were slightly higher than those in domestic pigs carrying the spontaneous *IGF2* intron 3 mutation [3] (4%). In addition, heavier livers (female founder: 27.6% and female F4: 13.1%) were observed in all female groups for *ZBED6*^-/-^ pigs (*p* < 0.05), whereas in male F4 pigs, the liver weight had no difference between WT and *ZBED6*^-/-^ pigs (**Fig 2A–2C**). The backfat thickness of *ZBED6*^*-*/-^ pigs in all three groups decreased compared with that of WT pigs (**S1C Fig**). Interestingly, the weights of the body, carcass, pigskin, lungs and kidneys were not different from those of WT pigs (**S3 Table**). In conclusion, *ZBED6*^-/-^ pigs have more muscle mass and larger internal organs.

### *ZBED6*^-/-^ pigs show muscle hypertrophy and less intramuscular lipid deposition

We further investigated whether the increased lean meat mass is caused by muscle hypertrophy. Hematoxylin and eosin (H&E) staining analysis revealed significantly thicker fibers in both the GM and myocardium of *ZBED6*^-/-^ pigs than that of WT controls (**Fig 2C**). Statistical analysis of the area measurements confirmed an ~128% increase in the fiber area of the *ZBED6*^-/-^ GM (**Fig 2D**). These experiments suggest that increased muscle mass and heart growth in *ZBED6*^-/-^ pigs mainly resulted from muscle fiber hypertrophy. Further oil red O staining showed fewer lipid droplets (**Fig 2E**) and at least 50% less triglyceride content (**Fig 2F**) in the GM of *ZBED6*^-/-^ pigs than in that of WT pigs, indicating reduced intramuscular lipid deposition.

### *ZBED6*^-/-^ increases *IGF2* mRNA expression in most tissues except liver

**Fig 3:**
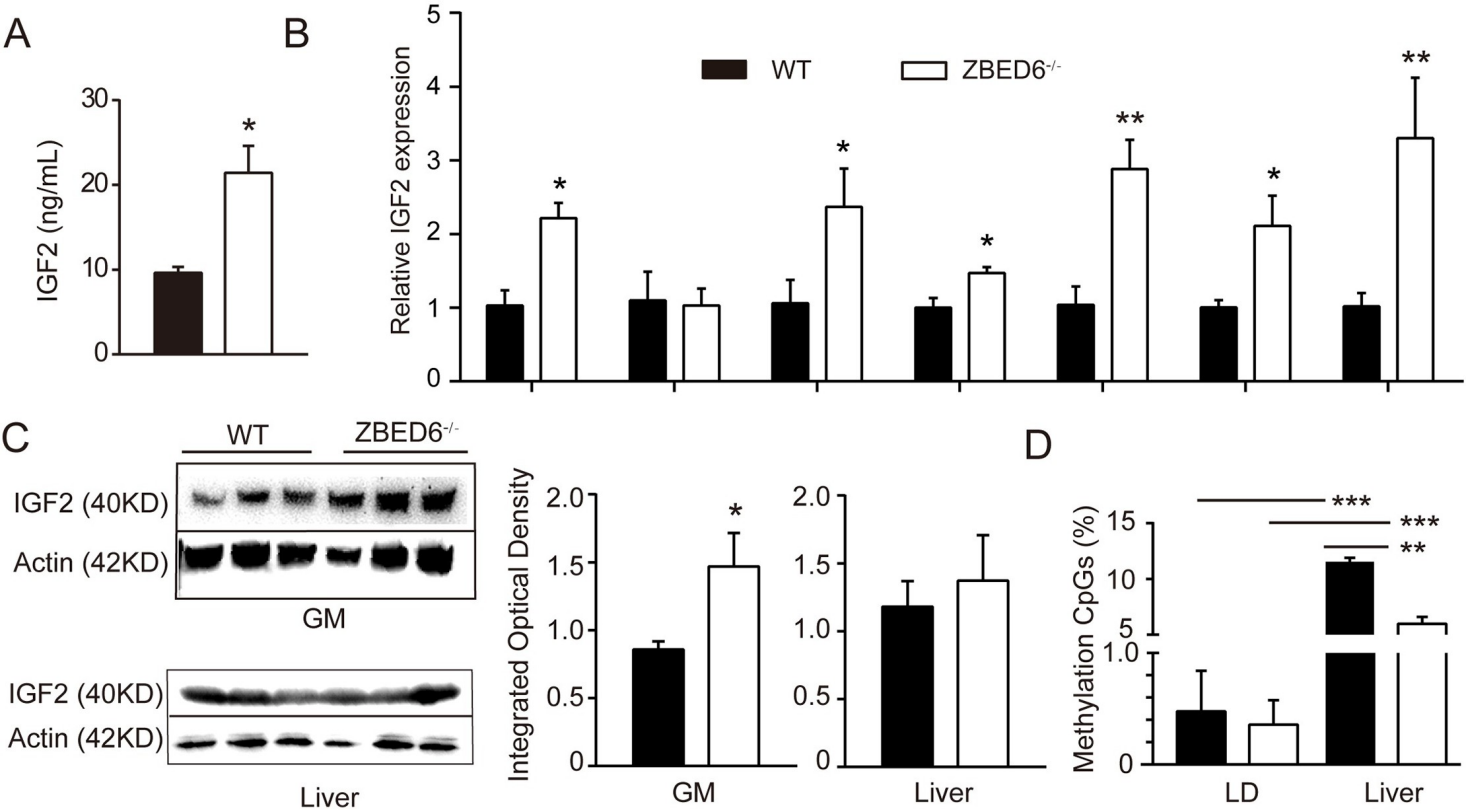
Increased levels of *IGF2* in multiple tissues except liver in WT and *ZBED6*^-/-^ pigs (n = 3). Gastrocnemius muscle (GM), *longissimus dorsi* (LD). **(A)** Serum concentrations of IGF2 in WT and *ZBED6*^-/-^ pigs at 3 months **(B)** qPCR analysis of *IGF2* mRNA in multiple tissues from *ZBED6*^-/-^ pigs. **(C)** Western blot analysis of IGF2 in GM and liver of *ZBED6*^-/-^ pigs. Results of western blot were quantified by Iamge J. The relative levels of IGF2 protein in GM and liver were plotted. **(D)** Percentage methylation around *IGF2*-intron 3–3072 (GCTCG) of LD and liver in WT and *ZBED6*^-/-^. The results are the means ± SEMs. *p < 0.05; **p < 0.01, *** *p* < 0.001, Student’s t test.

The serum IGF2 protein level of *ZBED6*^-/-^ pigs at three months was 2-fold higher than that of WT pigs (WT: *ZBED6*^-/-^ = 9.6: 21.4 ng/mL) (**Fig 3A**). qPCR analysis of *IGF2* expression showed increased *IGF2* mRNA levels in all tested tissues except liver from *ZBED6*^-/-^ pigs (**Fig 3B**). Western blot analysis with an anti-IGF2 antibody indicated that IGF2 protein content was increased in the GM of *ZBED6*^-/-^ pigs but kept unchanged in the liver (**Fig 3C**). These results suggest that increased muscle mass and heart growth in *ZBED6*^-/-^ pigs resulted from the upregulation of *IGF2* expression. We used bisulfite sequencing to examine the 56 CpG dinucleotides within a 345-bp fragment of this *ZBED6*-binding region in both skeletal muscle and in liver (**Figs 3D and S2**). We found on average that the methylation of CpGs in this 345-bp sequence is approximately 20-fold higher in liver than in muscle, which may explain the prevention of the ZBED6 interaction at the *IGF2* locus in the pig liver [3].

### *ZBED6* regulates many muscle regulators other than *IGF2*

**Fig 4:**
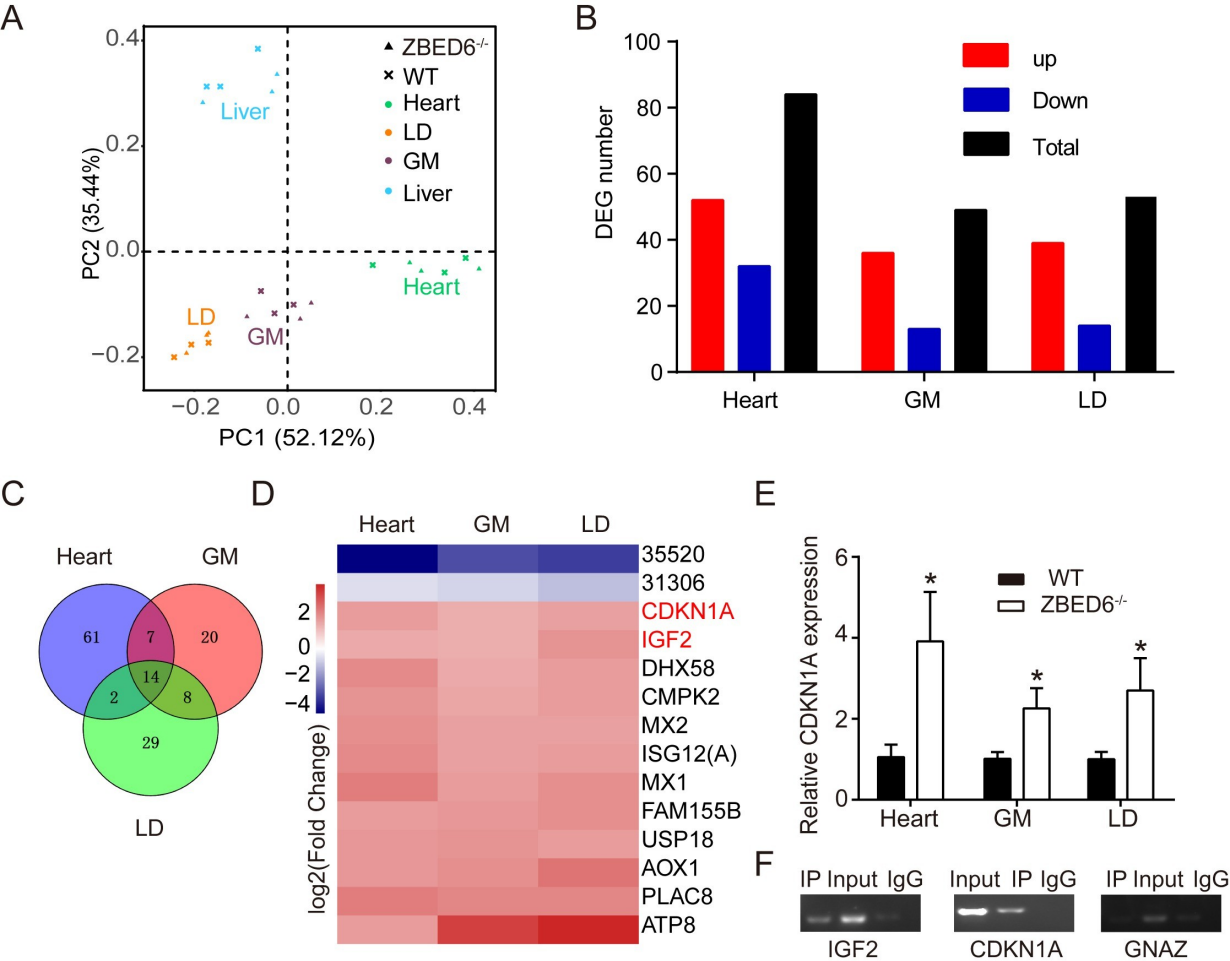
The regulation of *ZBED6* in the muscle. (n = 3). Gastrocnemius muscle (GM), longissimus dorsi (LD). **(A)** PCA of the expressed genes of multiple tissues in WT and *ZBED6*^-/-^ pigs. **(B)** Number of DEGs in heart, GM and LD in *ZBED6*^-/-^ pigs. **(C)** Overlap DEGs in heart, GM and LD. **(D)** Heatmap of common 14 DEGs in heart, GM and LD. 35520 and 31306 are two ensemble ID, ENSSSCG00000035520 and ENSSSCG00000031306. The genes in red type contain at least one ZBED6 ChIP-seq peak. **(E)** qPCR analysis of *CDKN1A* mRNA in the heart, GM and LD from *ZBED6*^-/-^ pigs. **(F)** ChIP-PCR analysis in gastrocnemius muscle of ZBED6 occupancy at the binding sites of *IGF2*, *CDKN1A* and *GNAZ*. The results are the means ± SEMs. *p < 0.05; **p < 0.01, *** *p* < 0.001,Student’s t test.

To explore the transcriptional changes caused by the disruption of *ZBED6*, we conducted RNA-seq analysis of four tissues, including the heart, gastrocnemius muscle, *longissimus dorsi* and liver, in *ZBED6*^-/-^ and WT pigs, with three biological replicates for each group (a total of 24 samples). Using the Illumina HiSeq 2500 platform, we generated approximately 39 to 57 million 150-bp paired-end reads and mapped 90% ~ 95% of the paired reads for each sample (**S4 Table**). PCA of these 24 samples clearly defined that the expression profile is unique in the liver but similar in the heart, gastrocnemius muscle and *longissimus dorsi* (**Fig 4A**). Differential expression analysis revealed 25,480 expressed genes and 50 to 85 differentially expressed genes (DEGs) across four tissues between the *ZBED6*^-/-^ and WT samples (**Fig 4B and S5–S8 Tables**), showing that *ZBED6* transcripts had no significant expression changes, and that the expression of Zinc finger CCCH domain-containing protein 11A (*ZC3H11A*), harboring *ZBED6* in its first intron, was not affected by *ZBED6* inactivation (**S3A–S3B Fig**). To confirm the accuracy of DEG identification based on RNA-seq, 8 DEGs were randomly chosen for qPCR validation, resulting in excellent agreement with the RNA-seq data (**S3C Fig**). Based on ZBED6 ChIP-seq peaks in C2C12 of mouse [4], 5 of 84 DEGs in the heart, 5 of 49 DEGs in the gastrocnemius muscle, 7 of 53 DEGs in the longissimus dorsi and 3 of 51 DEGs in the liver between the genotypes were found to contain at least one *ZBED6* ChIP-seq peak (GCTCG or CGAGC) (**S5–S8 Tables**). To identify genes containing at least one *ZBED6* binding site during muscle growth, we overlapped the DEGs of three muscle tissues and found fourteen common DEGs in the heart, gastrocnemius and *longissimus dorsi* between *ZBED6*^-/-^ and WT pigs (**Fig 4C**). Two of these fourteen DEGs—*IGF2* and cyclin-dependent kinase inhibitor 1A (*CDKN1A*)—contained at least one *ZBED6* ChIP-seq peak (GCTCG or CGAGC) [4]. Heatmap analysis with the log2(Fold Change) of DGEs between *ZBED6*^-/-^ and WT pigs and qPCR validation further showed that, other than the *IGF2*, *CDKN1A* was also consistently upregulated in *ZBED6*^-/-^ heart, gastrocnemius muscle and longissimus dorsi (**Fig 4D–4E**), which was identified as a candidate regulator. To elucidate whether *ZBED6* regulates *CDKN1A* in pigs, ChIP-PCR with anti-ZBED6 antibody was performed in pig gastrocnemius muscle. ZBED6 binding sites at the *IGF2* locus were ChIP down as expected and the negative control *GNAZ* showed no binding of ZBED6, both of which indicating our ChIP experiment was reliable (**Fig 4F**). According to the potential binding regions at the transcription start sites (TSS) of *CDKN1A* [9], we amplified upstream of *CDKN1A* after the chromatin immunoprecipitation with anti-ZBED6 antibody and confirmed that ZBED6 indeed directly binds to *CDKN1A* (**Fig 4F**). These results suggested that *ZBED6* regulates muscle growth via multiple target genes.

### Identification of putative *ZBED6* downstream targets in liver tissue

**Fig 5:**
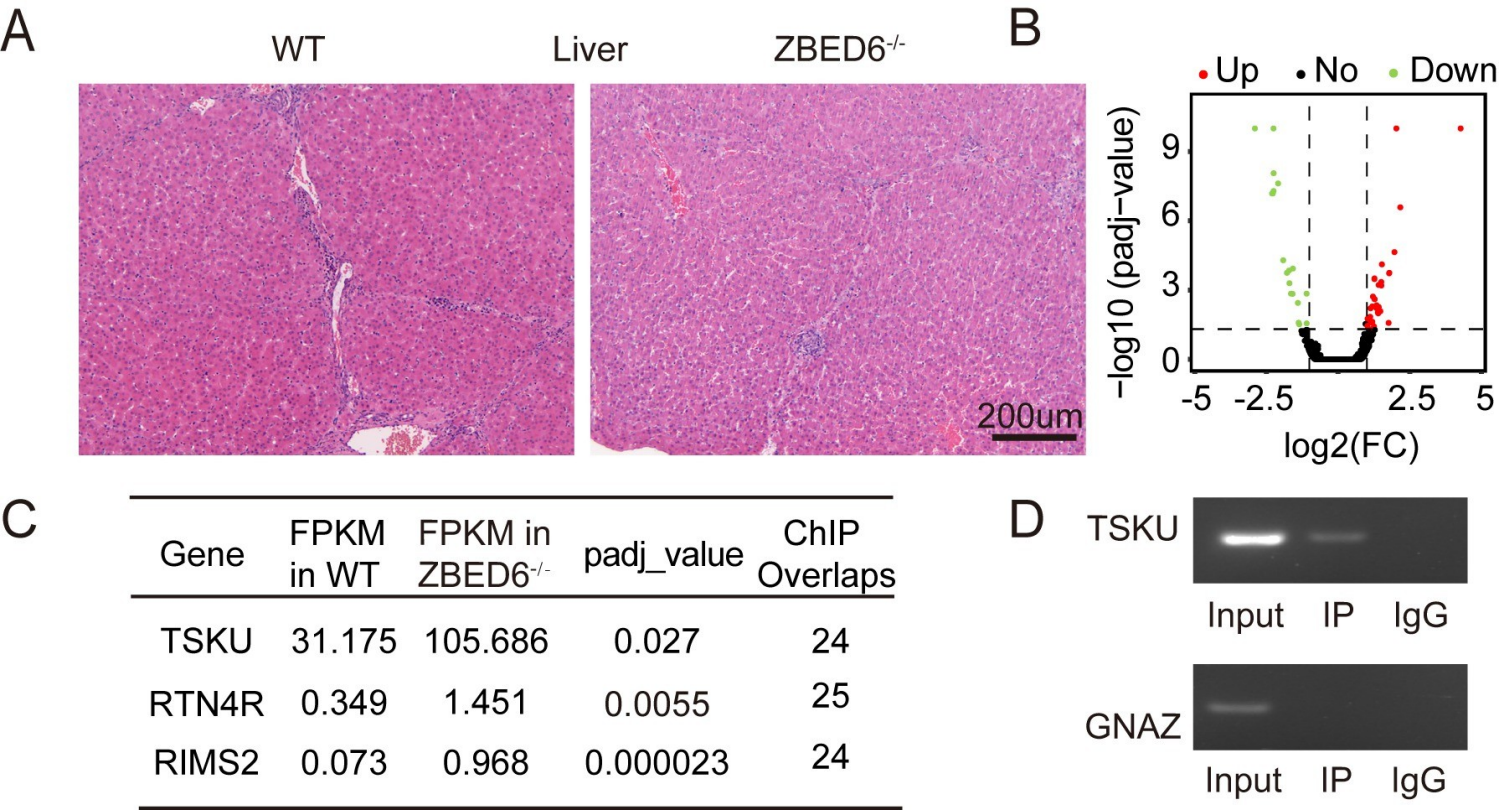
The regulation of *ZBED6* in the liver. (n = 3). **(A)** Haematoxylin and eosin (H&E) staining of the liver for WT and *ZBED6*^-/-^ 3-month-old pigs. **(B)** Volcano plot showing 51 DEGs of liver between WT and *ZBED6*^*-*/-^. **(C)** Three *ZBED6* targets of liver DEGs. ChIP overlaps = Number of overlapping extended reads. **(D)** ChIP-PCR analysis in liver of *ZBED6* occupancy at the binding sites of *TSKU* and *GNAZ*.

We noticed that loss of *ZBED6* did not affect hepatic *IGF2* expression, despite a 13.1% increase in liver weight (**Figs 2A–2B and 3B–3C).** H&E staining analysis revealed that the livers of *ZBED6*^-/-^ pigs had no structural difference compared with those of WT pigs (**Fig 5A)**. These results suggest that the effect of *ZBED6* on liver growth is mediated through targets other than *IGF2*. It is worth noting that *IGF2* mutant pigs, which have intact ZBED6 function and high *IGF2* mRNA expression, do not show any increased liver growth [3], supporting our conclusion that this effect is caused by other *ZBED6* target(s). Liver transcriptome analysis was performed to investigate putative *ZBED6* target genes other than *IGF2*. We identified 51 DEGs in the liver between *ZBED6*^-/-^ and WT pigs (**Fig 5B and S8 Table**), and *TSKU*, *RTN4R* and *RIMS2* of these DEGs were found and examined as *ZBED6* target gene according to the ChIP-seq of murine C2C12 cells [4] (**Fig 5C**). Among these three genes, *tsukushi*, *small leucine rich proteoglycan* (*TSKU*), with highest FPKM values in the liver tissue and a ZBED6 ChIP-seq peak, was *hepatokine* and identified as a candidate liver regulator. Encoding a small signaling molecule, the *TSKU* gene takes participates in liver growth and different developmental processes in vertebrates [15,16]. ChIP-PCR confirmed that ZBED6 indeed directly binds to *TSKU* (**Fig 5D**). These results suggest that *ZBED6* may affect liver growth by controlling targets other than *IGF2*, such as, *TSKU*.

## Discussion

The ZBED6-*IGF2* interaction is essential for controlling *IGF2* expression and muscle development postnatally in placental mammals [3,10,12]. The well-recognized *IGF2* intron 3–3072 mutation, abrogating the *ZBED6* interaction and thus increasing muscle growth, has been successfully selected in commercial lean pig breeds [3]. However, Chinese indigenous pigs still carry the wild-type allele and thus exhibit fat body composition.

Using CRISPR-Cas9, we successfully acquired five healthy *ZBED6*^-/-^ piglets from surrogate sow J11. The founder animals carrying a one-base pair deletion at position 1320-T of *ZBED6* developed and reproduced normally but showed postnatal overgrowth of the muscles, heart and liver compared with WT controls. Considering that the founders were derived from zygotes transferred to surrogate sows, while WT controls were born from natural mating, it is possible that this difference also contributed to the difference in phenotypes. To evaluate the effect of editing *ZBED6* more rigorously, the phenotypic data of female F4 pigs (n = 5:6) and male F4 pigs (n = 3:3) were added to verify that growth differences in muscles and internal organs between the WT and *ZBED6*^-/-^ pigs were actually due to the knockout of *ZBED6*. The observed effects of *ZBED6* knockout on muscle hypertrophy and heart growth were clearly more pronounced than those in domestic pigs carrying the *IGF2* intron 3 mutation and greater than those in *IGF2* knockin Bama pigs [3,11] (**Table 1**). Although the difference could be caused by breed-specific effects or experimental system errors, this finding suggests that the ZBED6-*IGF2* interaction is not the only essential axis in regulating muscle and heart. Similar to IGF2 mutant pig, the female and male F4 pigs also showed body weights similar to those of the WT pigs. The explanation of this could be that *ZBED6*^-/-^ pigs had more muscle mass and larger internal organs, while the backfat thickness of *ZBED6*^-/-^ pigs was decreased significantly in pigs. The study of fat was not the key point in this manuscript, but we will continue the investigation in the future. However, the effect on liver growth showed the sex difference in pig, a more pronounced increased liver in females than in males, which is also observed in *ZBED6* KO mice. A possible explanation for this sex difference is that the male pig in this study were not castrated, and phenotypic consequences of *ZBED6* knockout in male pig may have been masked by the action of testosterone. Overgrowth of liver was only showed in *ZBED6* KO mice, whereas *IGF2* KI mice and *IGF2* mutant pig showed no significant changes (**Table 1**) [3,10,11], supporting our conclusion that liver overgrowth in *ZBED6*^-/-^ pigs is independently from ZBED6-*IGF2* axis and likely occurs through the cooperative action of additional *ZBED6* target genes. The observed *IGF2* upregulation is consistent with the postnatal overgrowth of all tested organs except the liver in ZBED6^-/-^ pigs, suggesting ZBED6-*IGF2* axis has a major effect on the growth of muscle. Our findings provide reliable evidence not only for the importance of the ZBED6-*IGF2* axis in Chinese indigenous pigs but also for some new ZBED6 target genes affecting muscle and organ development of placental mammals. Loss of ZBED6 causes no difference in liver *IGF2* expression but marked changes in liver weight, thus strongly suggesting the existence of additional ZBED6 targets other than *IGF2*. This hypothesis was further confirmed by transcriptome analysis of four different tissues, including heart, gastrocnemius muscle, *longissimus dorsi* and liver. Comparative transcriptome analysis revealed a few new direct targets of ZBED6 in liver and muscle development regulation.

**Table 1:** The comparison of *ZBED6* KO and *IGF2* KI model in pigs and mice.

		Bama pig		Large white pig[1,3]	mice[10]		
genetic modification		ZBED6 KO (pigs in this study)	IGF2 KI[11]	IGF2 mutant	IGF2 KI	ZBED6 KO	
general	reproduction	normal	normal	normal	normal	normal	
	body weight	-	25% ↑	-	20% ↑	15% ↑	
	lean rate	6.6% ↑	NA	4% ↑	NA	NA	
	heart weight	17.2% ↑	NA	8% ↑	15% ↑	mild ↑	
	liver weight	13.1% ↑	NA	-	-	20–25% ↑	
	fat	10.4% ↓(BFT)	mild ↓(BFT)	20% ↓(BFT)	- (GWD)	- (GWD)	
	serum IGF2	2-fold ↑	NA	-	8 fold ↑	8 fold ↑	
muscle	hypertrophy	GM and myocardium	LD	NA	NA		
	fiber area	128% ↑	40% ↑				
	triglyceride	50% ↓	NA				
IGF2	heart	1.5-fold ↑	NA	1-fold ↑	4 fold ↑		8 fold ↑
	muscle	1.5-fold ↑ (GM)	2-fold ↑	1.2-fold ↑	15 fold ↑ (TA)		20 fold ↑ (TA)
		2.5-fold ↑(LD)					
	kidney	2 fold ↑	NA	NA	10 fold ↑		30 fold ↑
	liver	-	NA	-	NA		NA
IGF2 methylated	liver	10%	NA	26%	NA		
	muscle	0.50%	1.25%	3.40%			

BFT: back fat thickness, GWD: gonadal white adipose, TAW: tibialis anterior weight, GM: gastrocnemius muscle

*ZBED6* inactivation in pigs leads to striking upregulation of serum *IGF2* levels during the postnatal stage (approximately two-fold increase). Although an even more dramatic increase in serum *IGF2* levels (eight-fold) was seen in both *IGF2* KI and *ZBED6* KO mice, serum *IGF2* content was not changed in domestic pigs carrying *IGF2* mutations (**Table 1**) [3,10], suggesting that there are species-differences as regards to the regulation of serum *IGF2* levels which requires further investigations. The liver is the main endocrine source of *IGF2* in postnatal life [17], but surprisingly, the present study found that *ZBED6* inactivation did not result in significant changes in *IGF2* mRNA expression in pig liver when quantified by RNA-seq, qPCR analysis and western blot, which had been proved in our *ZBED6* heterozygous samples [18]. Further investigation of the DNA methylation status suggested that the ZBED6-*IGF2* interaction in the liver was hampered by the 20-fold higher methylation at the *IGF2* CpG island than that in muscle, consistent with a previous report in domestic pigs carrying the *IGF2* mutation (**Table 1**) [3]. Therefore, autocrine/paracrine IGF2 activity, for example, muscle *IGF2* expression, can greatly influence circulating IGF2 levels at the postnatal stage.

The differential expression analysis indeed revealed a number of new ZBED6 target genes controlling liver and muscle development in placental mammals, such as *TSKU*, and *CDKN1A*. *TSKU* is involved in diverse developmental processes mediated by the *FGF*, *TGF*-β and Wnt pathways, for example, bone growth, development of the inner ear, Xenopus germ layer formation, and the mouse hair cycle [15,19–21]. The role of *FGF*, *TGF*-β and Wnt signaling in liver development has been reviewed elsewhere [16]. As a result, we propose that *ZBED6* may regulate liver growth partially by the interactions of *TSKU* with the *FGF*, *TGF*-β and Wnt pathways. In addition to *IGF2*, *CDKN1A* which contained at least one *ZBED6* ChIP-seq peak, was also found upregulated in *ZBED6*^-/-^ heart, gastrocnemius muscle and longissimus dorsi. *CDKN1A* (also known as p21) is a negative regulator in the cell cycle and is required in skeletal muscle regeneration [22–24] and cardiac development [22–25]. Therefore, in addition to its major effects on *IGF2*, ZBED6 may mildly regulate muscle development by controlling *CDKN1A* expression. These discovery are potentially interesting for future investigations using the *ZBED6* KO pig model.

## Materials and methods

### Ethics statement

All animal experiments were performed in accordance with the regulations and guidelines established by the Animal Care Committee of the Beijing Academy of Agricultural Sciences (Approval number: IAS2019-60).

### Animals

Bama miniature pigs were provided by the pig farm affiliated with the Institute of Animal Science, Chinese Academy of Agricultural Sciences (CAAS), Beijing, China. The pigs had ad libitum access to a commercial pig diet and water throughout the experimental period.

### A CRISPR/Cas9-mediated, nonhomologous end-joining-independent integration strategy

The methods used for knocking out *ZBED6* in Bama miniature pigs were similar to those in previous studies [26]. Four single-guide RNAs (sgRNAs) were designed according to the sequence of *ZBED6* using Optimized CRISPR Design tools (http://crispr.mit.edu/) and synthesized by GBI (Guangzhou, China). Four microlitres synthesized sgRNAs were annealed to double-stranded DNA and then cloned into 1 μL (50–100 ng) plasmid pX330-Cas9 (China Agricultural University, Beijing, China) at 16°C for 1 h to form the sgRNA-pX330-Cas9 plasmids. Four micrograms the correct constructs were purified using the EndoFree Plasmid Maxi Kit (Qiagen) and then transfected into 10^6^ porcine fetal fibroblasts (PFFs) by electroporation using the Amaxa Nucleofector Kit (Lonza, Germany). The transfected cells were cultured in DMEM (Gibco, Grand Island, NY, USA) containing 20% FBS (Gibco, Grand Island, NY, USA) for 48 hours at 37°C with 5% CO_2_, and genomic DNA was extracted using Promega Wizard Genomic Purification (Promega, USA). The DNA was amplified (Bio-Rad, USA) using ZBED6-1 primers flanking the sgRNA targeting locus. The PCR conditions were 95°C for 5 min; 95°C for 30 s, 60°C for 30 s, and 72°C for 25 s for 32 cycles; 72°C for 10 min; and a hold at 4°C. The 429-bp PCR product was purified using a NucleoSpin Gel and PCR Clean-up Kit (Macherey-Nagel, Germany). The purified fragments were TA cloned to evaluate the mutagenic efficacy of four sgRNAs by Sanger sequencing (BGI, Guangzhou, China).

Using the Amaxa Nucleofector Kit (Lonza, Germany), 4 μg sgRNA3-pX330-Cas9 plasmid with the highest efficiency (sgRNA3) were transfected into 10^6^ PFFs, and the transfected cell number was calculated using an automated cell counter (Millipore, USA). Transfected cells (15–100) were grown in 10-cm dishes for 15 d to isolate single cell clones, which were then selected and propagated until full confluency in each well of 48-well plates to screen the positive cell clones by Sanger sequencing using the *ZBED6*-1 primer (BGI, Guangzhou, China).

Somatic cell nuclear transfer (SCNT) was performed with the method described previousl [27]. Oocytes were enucleated by aspirating the polar body. Three positive single clones were selected as donor cells for SCNT to be injected into the perivitelline space of the oocytes. Fusion and activation were performed by an electrofusion instrument (ECM, BTX, USA) using two successive DC pulses at 1.2 kV/cm for 30 μs. A total of 581 reconstructed embryos were transferred into the oviducts of three surrogate sows, and the cloned pigs were delivered at full term by natural birth. The genotyping of *ZBED6* mutagenesis was assessed at the targeted site by PCR-based assays.

### Off-target analysis

Although the CRISPR/Cas9 system has the advantage of high gene targeting efficiency in pigs, undesired off-target effects are still a major concern [28]. To determine whether off-target mutagenesis occurred in the *ZBED6*^-/-^ piglets, 10 off-target sequences (OTSs) for sgRNA3 were identified by the online CRISPR design tool (http://crispr.mit.edu/) and tested in the *ZBED6*^*-/-*^ pigs by Sanger sequencing with specific primers (**S2 Table**).

### Pig housing and cross

Five *ZBED6*^-/-^ female founders were maintained together with five female WT controls derived from natural mating of similar age to the founders. For mating, a 6-month-old female founder (#178) was crossed with a WT male pig (6 months old). Then, adult male and female piglets of F1, F2 and F3 that carried heterozygous mutations were crossed. Finally, F4 pigs were born by natural delivery. Female founders (three WT and three *ZBED6*^-/-^) at 8 month of age, female F4 pigs (five WT and six *ZBED6*^-/-^) and male F4 pigs (three WT and three *ZBED6*^-/-^) at 5 month of age were all tested for slaughtering experiment.

### Slaughtering experiment

The body weights of founder and F4 pigs were measured every month after birth. Blood samples of ZBED6^-/-^ pigs at 3 months were collected to measure the serum IGF2 level using a porcine IGF2 ELISA Kit (MLBio, Shanghai Enzyme-linked Biotechnology Co., Ltd.). The pigs were euthanized and slaughtered to evaluate meat production performance. The hairs, internal organs, head and hoofs were removed and weighed from each pig, and then the carcass weight was measured. Bone, skin and lean mass were dissected from the left carcass and weighed. The lean meat rate was determined by the ratio of lean meat weight and carcass weight of the left carcass. Tissue samples of the heart, longissimus dorsi, gastrocnemius muscle and liver were collected for different purposes: one-third were preserved in RNAlater reagent (Thermo Fisher Scientific, USA) for RNA extraction, one-third were fixed in 4% paraformaldehyde fixation solution (ABI, USA) to prepare paraffin sections, and one-third were preserved in liquid nitrogen for protein expression quantification.

### Genotyping of *IGF2* intron 3–3072 and *ZBED6* mutagenesis in piglets

*ZBED6* and *IGF2* genotyping primers flanking sgRNA3 and nucleotide 3072 of *IGF2*-intron 3 were designed to genotype piglets. To genotype the genomic DNA that was isolated from the ear skin biopsy of the piglets., DNA was amplified (Bio-Rad, USA) with 94°C for 5 min; 94°C for 30 s, 60°C for 30 s, and 72°C for 30 s for 35 cycles; 72°C for 5 min; and a hold at 4°C using the *ZBED6* (*ZBED6*-1) and *IGF2* (*IGF2*-1) genotyping primers (**S9 Table**). PCR products were purified using a NucleoSpin Gel and PCR Clean-up Kit (Macherey-Nagel, Germany). Purified PCR products were cloned for genotyping by Sanger sequencing (BGI, Guangzhou, China).

### Histological analysis

Liver, heart and gastrocnemius muscle tissues were prepared for histological sectioning following the instruction manual. Briefly, tissues were fixed in a 4% paraformaldehyde solution and embedded in paraffin wax. The paraffin-embedded tissue blocks were sectioned into 5-μm-thick slices using a Leica RM2255 Automated Rotary Microtome (Germany), which were stained by HE and oil red O staining. Morphological observations at 100x and 50x magnification were conducted with an Olympus BX51 microscope (Olympus, Japan). Five fields of each sample were observed, and the muscle fiber-related parameters were assessed by Image-Pro plus 6.0 (Media Cybernetics, Bethesda, USA). Triglycerides of gastrocnemius muscle were extracted and quantified by AceChem TG Kit (Technology built, Nanjing, China).

### Bisulfite-based methylation analysis

Bisulfite sequencing was performed as described [3]. Three *ZBED6*^*-/-*^ and three WT pigs were tested. Genomic DNA was extracted with a Wizard Genomic DNA Purification Kit (Promega, USA) from *longissimus dorsi* and liver tissues of *ZBED6*^*-/-*^ and WT pigs, followed by bisulfite treatment with an EZ DNA Methylation-Gold Kit (Zymo Research, USA). A 345-bp fragment centered around *IGF2* intron 3–3072 (GCTCG) was amplified (Bio-Rad, USA) using a two-step PCR with the following primers: PCR1-F, 5’ -TTTYGGGGATTGTTGAAGT- 3’, PCR1-R, 5’ -AAACAATCCCCAATAA- 3’, PCR2-F, 5’ -GGGGATTGTTGAAGTTTT- 3’, and PCR2-R, 5’ -CTTCTCCTACCACTAAA- 3’. PCR products were cloned and sequenced (Qingke, Beijing).

### RNA sequencing

RNA of three female founder WT and *ZBED6*^-/-^ pigs were extracted using an RNeasy Mini Kit (QIAGEN, Germany). RNA concentration and quality were determined using an Agilent 2100 Bioanalyzer (USA). Samples with a RIN value greater than 7.0 were used for downstream real-time PCR quantification and RNA-seq analysis. An NEBNext Poly(A) mRNA Magnetic Isolation Module was used to isolate poly(A) mRNA from total RNA, and an NEBNext Ultra RNA Library Prep Kit was used to prepare the RNA-seq libraries. Transcriptome sequencing was performed on an Illumina HiSeq 2500 platform at Berry Genomics Company (Beijing, China) for 150 cycles in paired-end mode. The pig genome assembly Sscrofa11.1 was downloaded (ftp://ftp.ensembl.org/pub/release-101/fasta/sus_scrofa/dna/Sus_scrofa.Sscrofa11.1.dna.toplevel.fa.gz). The clean reads were mapped to the pig reference genome using Hisat2 software (version 2.1.0) with default parameters. The featureCounts program was used to calculate gene expression, and the DEseq2 package was used to analyze differences between WT and ZBED6^-/-^ group. Genes with q value<0.05 and FoldChange> 2 were defined as differentially expressed genes (DEGs).

### Principal component analysis (PCA)

The PCA was performed using gmodels in R (version 3.1.3, http://cran.r-project.org/)). and the FPKM values for all of the annotated transcripts from the four tissue transcriptomes.

### Chromatin immunoprecipitation (ChIP)-PCR analysis

ChIP was performed in native conditions [29]. Briefly, freshly snap-frozen tissues were treated with 1% formaldehyde in medium for 18 min and neutralizing with glycine (AMRESCO, USA) for 5 min at room temperature. After two washes with ice-cold PBS (Gibco, Grand Island, NY, USA) containing protease inhibitors, the tissues were smashed and resuspended in SDS lysis buffer (Beyotime, China). After incubation for 20 min at 4°C, the lysates were sonicated 24 times (30 s each) (Bioruptor Sonication System, USA). An equal amount of chromatin was immunoprecipitated at 4°C overnight with at least 5ug of the following antibodies: anti-ZBED6 antibody (HPA068807, ATLAS) and normal mouse IgG antibody (2729S, CST). Immunoprecipitated products were collected after incubation with Protein G agarose beads (ThermoFisher Scientific, USA). The beads were washed, and bound chromatin was eluted in ChIP Elution Buffer. Add 20 microliters 5M NaCl to the combined eluates and reverse histone-DNA crosslinks by heating at °65 for 4 hours. And then added RNase A (TIANGEN, China) for 30 min at 37°C, and proteins were digested with Proteinase K (TIANGEN, China) for 2h at 65°C. Coprecipitated DNAs were purified using a ChIP DNA Clean &Concentrator purification spin column (ZYMO, Irvine, California, USA) and eluted in 20 μL dlution buffer. The ZBED6 binding site, *IGF2*, *CDKN1A*, *TSKU* and *GNAZ* was evaluated using PCR and normalized by total chromatin (input). The *GNAZ* was selected as a negative control gene because it contained no ChIP-seq binding signal of ZBED6[4]. Normal mouse IgG was used as the negative control, the primers are described in **S9 Table**. The PCR conditions were: 1 cycle at 95°C for 5 min followed by 33 cycles at 95°C for 30 s, 58–59°C for 30 s, and 72°C for 30s. The PCR products were then electrophoresed on 1.5% agarose gels stained with GelGreen (TIANGEN, China).

### Western blotting

Gastrocnemius muscle and liver were dissected and frozen immediately in liquid nitrogen until use. Total proteins from tissue were extracted using a Total Protein Extraction Kit (Technology built, Nanjing, China). The proteins were subjected to Western blot analysis with the following antibodies: anti-ZBED6 antibody (HPA068807, 1:500; ATLAS), anti-IGF2 antibody (ABC504, 1:500; Merck-Millipore) and anti-Actin antibody (HRP-60004, 1:1000; Proteintech). The blots were developed using HRP-conjugated secondary antibodies. Picture were captured by imaging system (Alpha Innotech, Shanghai, China) and quantified by Image J software.

### Quantitative PCR

Total RNA was extracted using an RNeasy Mini Kit (QIAGEN, Germany). A PrimeScript RT Reagent Kit with gDNA Eraser (Takara, China) was used to generate cDNA from RNA. qPCR analysis was performed in ABI MicroAmp optical 96-well reaction plates on an ABI 7500 real-time PCR instrument (USA) using SYBR Premix Ex Taq (Tli RNaseH Plus; Takara). The data were normalized to the expression of the housekeeping genes GAPDH, 18S and β-actin. The primer sequences are listed in **S9 Table**. Relative gene expression was calculated using the comparative cycle threshold (2^-ΔΔCt^) method [30].

### Statistical analysis

The statistical data reported include results from at least three biological replicates. All statistical analyses were performed using GraphPad Prism 7 (GraphPad Software Inc., La Jolla, CA, USA). Values are presented as the mean ± standard error of the mean (SEM). The P values were determined by Student’s t test. Error bars indicate the SEM. *P < 0.05; **P < 0.01, *** *P* < 0.001.

## Supporting information

S1 Fig Phenotype of *ZBED6*^-/-^ pigs. **(A)** *IGF2* sequence of Bama pigs are 100% fixed for the wild-type allele (G) at *IGF2*-intron 3–3072. Underlined sequence (GCTCG) represent the binding sites of *IGF2* and *ZBED6*. **(B)** Body weight measurements of WT and *ZBED6*^-/-^ in female founder, female and male F4 pigs. *ZBED6*^-/-^ pigs showed similar weight with WT pigs starting from birth until six months. **(C)** Backfat thickness of WT and *ZBED6*^-/-^ in female founder, female and male F4 pigs. *ZBED6*^-/-^ pigs had thinner backfat thickness than WT pigs. Red points represent actual data of carcass traits.
(TIF)

S2 Fig Methylation around *IGF2*-intron 3–3072 (GCTCG) of longissimus dorsi (LD) and liver in WT and ZBED6^-/-^ pigs (n = 3). WT and ZBED6^-/-^ pigs allele contains 56 CpGs. Unfilled (blue) and filled (red) boxes represent unmethylated and methylated CpGs, respectively. The results of methylation showed approximately 20-fold higher in the liver than in the LD (liver:LD = ~10.0%:0.5%).
(TIF)

S3 Fig ZBED6 inactivation did not affect the fpkm of ZBED6 and ZC3H11A genes (n = 3). **(A-B)** The RNA-seq data of heart, liver, gastrocnemius muscle (GM) and longissimus dorsi (LD) tissues with three ZBED6^-/-^ and three WT pigs, did not reveal any altered transcriptional expression of ZBED6 and the host gene ZC3H11A after ZBED6 inactivation in any of the tissues studied. **(C)** qPCR and RNA-seq results of 8 DEGs in heart, gastrocnemius muscle (GM) and longissimus dorsi (LD) showed same trend.
(TIF)

S1 Table Summary of the ZBED6 gene editing (four sgRNAs sequences, efficiency of ZBED6 cloning mutation, and embryo transfer results). (PDF)

S2 Table Primer sequences information for sgRNA-3 potential off-target sites, and Sequenceing analysis of potential off-target sites of sgRNA-3. (PDF)

S3 Table Growth performance and slaughter indexes between WT and ZBED6^-/-^. (PDF)

S4 Table The quality analysis and genome mapping analysis of transcriptome sequencing. (PDF)

S5 Table The DEGs of heart between WT and ZBED6^-/-^ pigs. (PDF)

S6 Table The DEGs of gastrocnemius muscle between WT and ZBED6^-/-^ pigs. (PDF)

S7 Table The DEGs of *longissimus dorsi* between WT and ZBED6^-/-^ pigs. (PDF)

S8 Table The DEGs of liver between WT and ZBED6^-/-^ pigs. (PDF)

S9 Table Primer pairs for PCR, qRT-PCR and ChIP-PCR. (PDF)

S1 Data Raw Data for Figs 1–5, S1 and S3. (XLSX)
