# Supplementary figures and images for: Porcine ZBED6 regulates growth of skeletal muscle and internal organs via multiple targets
Source: PLoS Genet. 2021 Oct 28;17(10):e1009862. doi: 10.1371/journal.pgen.1009862 (PMC8577783; doi:10.1371/journal.pgen.1009862)

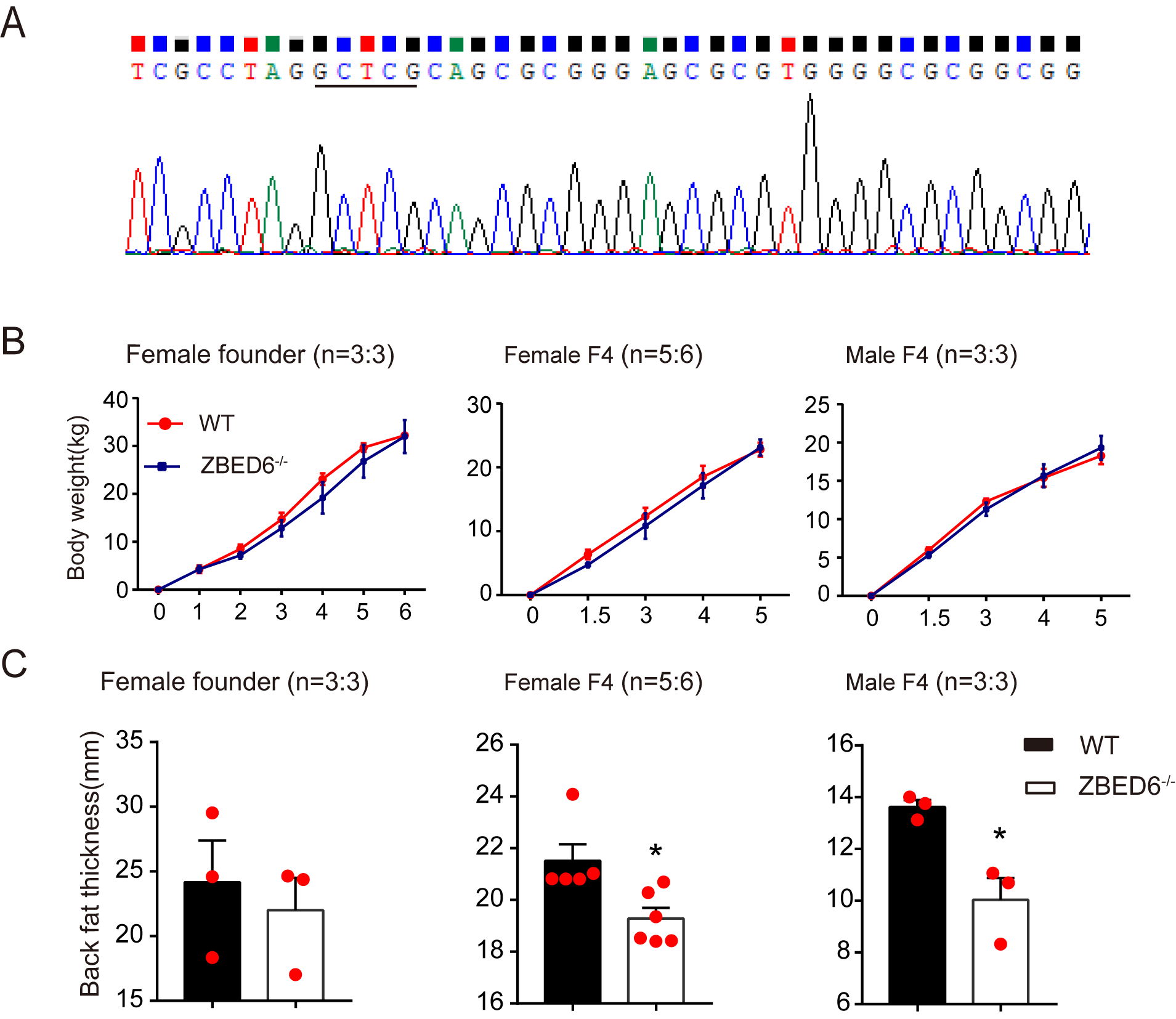

Supplement: S1 Fig — (A) IGF2 sequence of Bama pigs are 100% fixed for the wild-type allele (G) at IGF2-intron 3–3072. Underlined sequence (GCTCG) represent the binding sites of IGF2 and ZBED6. (B) Body weight measurements of WT and ZBED6-/- in female founder, female and male F4 pigs. ZBED6-/- pigs showed similar weight with WT pigs starting from birth until six months. (C) Backfat thickness of WT and ZBED6-/- in female founder, female and male F4 pigs. ZBED6-/- pigs had thinner backfat thickness than WT pigs. Red points represent actual data of carcass traits. (TIF) [file pgen.1009862.s001.tif]

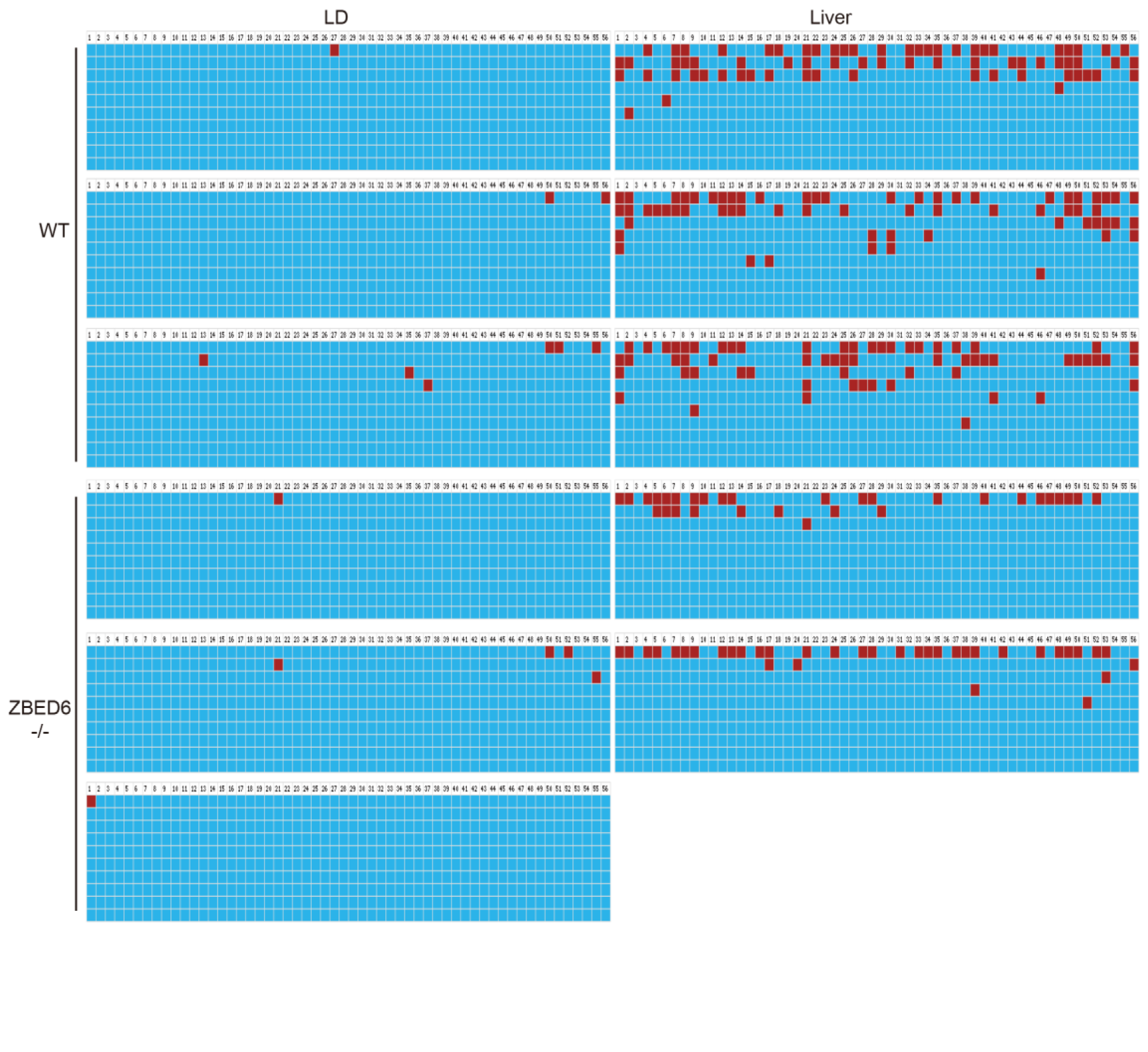

Supplement: S2 Fig — WT and ZBED6-/- pigs allele contains 56 CpGs. Unfilled (blue) and filled (red) boxes represent unmethylated and methylated CpGs, respectively. The results of methylation showed approximately 20-fold higher in the liver than in the LD (liver:LD = ~10.0%:0.5%). (TIF) [file pgen.1009862.s002.tif]

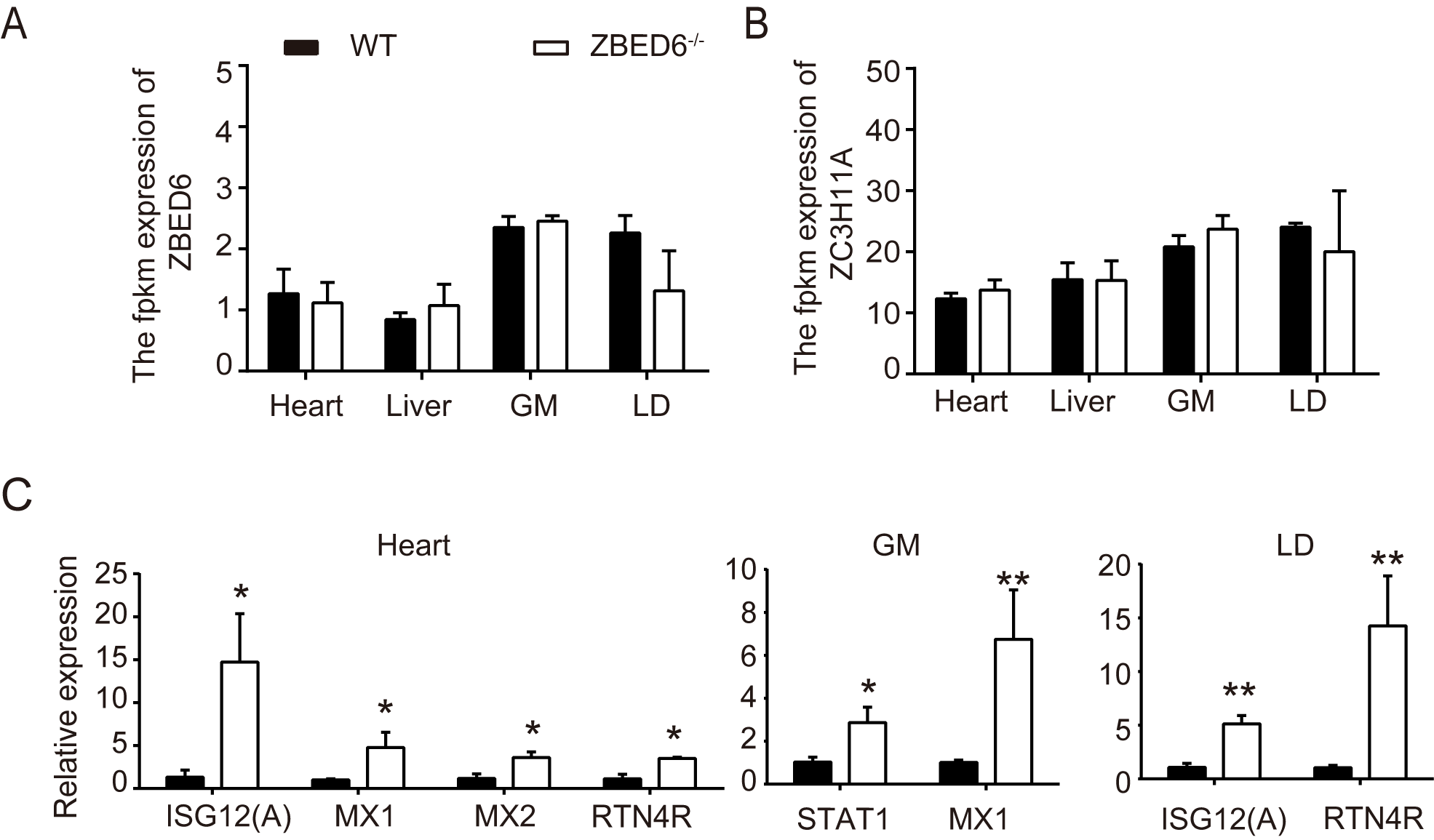

Supplement: S3 Fig — (A-B) The RNA-seq data of heart, liver, gastrocnemius muscle (GM) and longissimus dorsi (LD) tissues with three ZBED6-/- and three WT pigs, did not reveal any altered transcriptional expression of ZBED6 and the host gene ZC3H11A after ZBED6 inactivation in any of the tissues studied. (C) qPCR and RNA-seq results of 8 DEGs in heart, gastrocnemius muscle (GM) and longissimus dorsi (LD) showed same trend. (TIF) [file pgen.1009862.s003.tif]
