# Supplementary material for: Porcine ZBED6 regulates growth of skeletal muscle and internal organs via multiple targets
Source: PLoS Genet. 2021 Oct 28;17(10):e1009862. doi: 10.1371/journal.pgen.1009862 (PMC8577783; doi:10.1371/journal.pgen.1009862)
Supplement: S1 Table — (PDF) [file pgen.1009862.s004.pdf]

Four sgRNAs sequences designed to uniquely target ZBED6

sgRNA	sgRNA-1	sgRNA-2	sgRNA-3	sgRNA-4
sequence 5'→3'	agaagggtttgcgaattaag	gtaaggatttgggatctggg	agagcaaagacttccattgtg	gtatacctggc gggcaattt
gene knockout efficiency	0%	0%	7.50%	0%

Efficiency of ZBED6 cloning mutation

sgRNA	Number of cell colonies	Efficiency of wildtype	Efficiency of gene mutation	
			68%（39/57）	
sgRNA-3	57	32%（18/57）	Efficiency of biallelic mutation	Efficiency of allelic mutation
			49%（28/57）	19%（11/57）

Summary of embryo transfer results from gene-targeted PEFs

Pig ID surrogate	Donor cells	Embryos transferred	Pregnancy in 28 days	Piglets	KO	Survival piglets
J-10	Z17(+1 bp/+1 bp)	187	Yes	5	Yes	0
J-12	Z23(-1 bp/-1 bp)	212	Yes	5	Yes	1(#182)
J-11	Z28(-1 bp/-1 bp)	182	Yes	6	Yes	5 (#175,#176,#177,#178, #180)
Ttotal	3	581	3	16	16	6
