# Supplementary material for: Porcine ZBED6 regulates growth of skeletal muscle and internal organs via multiple targets
Source: PLoS Genet. 2021 Oct 28;17(10):e1009862. doi: 10.1371/journal.pgen.1009862 (PMC8577783; doi:10.1371/journal.pgen.1009862)
Supplement: S2 Table — (PDF) [file pgen.1009862.s005.pdf]

Primer sequences information for sgRNA-3 potential off-target sites

Number	Forward prime	Reverse primer	Amplicon length
1	GACCTAGCTCAGCAACTCCA	ACCTGCTCTTTTGGCCATTG	573
2	AGAACACACAAGTCACCATA	AAGAAGCAAGAGAGAGCCCC	643
3	GTTCTTTCCTGACCCTGGGA	AATACCAGCCCAAAGTCGGA	608
4	TTCTGTTCTAGGGGTCGCTG	GTGGCTCAGCGGTAATGAAC	566
5	GTAGCTCCTTCCCTCTGTGG	CCCCTATGTGTCCCCAAACT	565
6	ATCGACACTGGCAGAAGGAA	AGCAGCTTAGACACCCTGAG	620
7	ATCAGGCCCCGACCATATTC	TGATCCCGAACCCAACAGAT	667
8	GCATCACTAAGTCAGCCAGC	CTGATTAGACCCCTAGCCCG	630
9	GCCAGATTTGACTCAAGGGC	TGTTAAGGCAGAGGGGATGG	666
10	CTTTGGAGGTGGTGCTCAAC	ACAGGTGATTGCATGAGGGA	652

Sequenceing analysis of potential off-target sites of sgRNA-3

Number	Chromosome	Sequence of potential off-target site	Mismatch number	Off-target analysis
1	Chr9	AAGGAAAGAATTCCATTGTGCAG	3	WT
2	Chr10	TAACCAAGAATTCCATTGTGCGG	4	WT
3	Chr13	GAGGAAAAACTTACATTGTGAAG	3	WT
4	Chr6	CAGAAATGATTTCCATTGTGAGG	4	WT
5	Chr1	GAACAATTATTTCCATTGTGGAG	4	WT
6	Chr8	AAACAAATGCTTCCATTGTGCAG	4	WT
7	Chr1	GGTCAAAGATTCCCATTGTGCAG	4	WT
8	Chr9	TAGAAAAGATTTCCATGGTGTAG	4	WT
9	Chr4	GAGCGTGCACTTCCATTGTGCAG	4	WT
10	Chr16	GAACAAACAGTTTCATTGTGTGG	4	WT
