# Supplementary material for: Porcine ZBED6 regulates growth of skeletal muscle and internal organs via multiple targets
Source: PLoS Genet. 2021 Oct 28;17(10):e1009862. doi: 10.1371/journal.pgen.1009862 (PMC8577783; doi:10.1371/journal.pgen.1009862)
Supplement: S3 Table — (PDF) [file pgen.1009862.s006.pdf]

Growth performance and slaughter indexes between WT and ZBED6-/- female founder pigs													
Trait	body weight（kg）	carcass weight（kg）	slaughter rate（%）	left carcass weight（kg）	lean mass	lean meat rate（%）	pigskin weight (kg)	bone weight (kg)	back fat thickness(mm)	heart weight (kg)	liver weight (kg)	lung weight (kg)	kidney weight (kg)
WT Bama miniatures (n=3)	32.2±0.245	23.233±0.68	0.721±0.017	11.633±0.657	5.08±0.172	0.437±0.016	1.951±0.174	1.289±0.227	24.153±3.967	0.113±0.008	0.464±0.02	0.264±0.065	0.123±0.008
ZBED6-/- Bama miniatures（n=3）	35.533±2.766	25.577±2.106	0.72±0.006	13.267±1.236	6.466±0.543	0.488±0.014	1.791±0.073	1.446±0.049	22.015±3.967	0.145±0.012	0.592±0.051	0.281±0.012	0.122±0.028
p-value	0.215	0.264	0.903	0.226	0.041*	0.025*	0.357	0.455	0.269	0.048*	0.047*	0.778	0.969

Growth performance and slaughter indexes between WT and ZBED6-/- female founder pigs													
Trait	body weight（kg）	carcass weight（kg）	slaughter rate（%）	left carcass weight（kg）	lean mass	lean meat rate（%）	pigskin weight (kg)	bone weight (kg)	back fat thickness(mm)	heart weight (kg)	liver weight (kg)	lung weight (kg)	kidney weight (kg)
WT Bama	32	22.2	0.6938	11	4.852	44.109	2.23	1.534	29.5325	0.101	0.474	0.371	0.116
miniatures	32.6	24.1	0.7393	12.7	5.336	42.016	1.766	1.408	18.34	0.124	0.487	0.209	0.118
(n=3)	32	23.4	0.7313	11.2	5.052	45.107	1.858	0.926	24.5875	0.113	0.432	0.213	0.136
Mean	32.2	23.233	0.721	11.633	5.08	0.437	1.951	1.289	24.153	0.113	0.464	0.264	0.123
SEM	0.244	0.68	0.017	0.657	0.172	0.011	0.174	0.227	3.967	0.008	0.02	0.065	0.008
ZBED6-/-	39.6	28.8	0.7273	15.2	7.2	47.368	1.878	1.502	17.025	0.159	0.6	0.299	0.158
Bama	35.2	25	0.7102	12.8	6.53	51.016	1.818	1.468	24.3725	0.15	0.66	0.266	0.079
miniatures [illegible]	31.8	22.93	0.7211	11.8	5.668	48.034	1.678	1.368	24.6475	0.127	0.516	0.277	0.13
Mean	35.533	25.577	0.72	13.267	6.466	0.488	1.791	1.446	22.015	0.145	0.592	0.281	0.122

SEM	2.766	2.106	0.006	1.236	0.543	0.014	0.073	0.049	3.058	0.012	0.051	0.012	0.028
p-value	0.215	0.264	0.903	0.226	0.041*	0.025*	0.357	0.455	0.269	0.048*	0.047*	0.778	0.969

Growth performance and slaughter indexes between WT and ZBED6-/- female F4 pigs

Trait	body weight（kg）	carcass weight（kg）	slaughter rate（%）	left carcass weight（kg）	lean mass	lean meat rate（%）	pigskin weight (kg)	bone weight (kg)	back fat thickness(mm)	heart weight (kg)	liver weight (kg)	lung weight (kg)	kidney weight (kg)
WT Bama miniatures (n=5)	24.36±1.115	15.794±0.993	0.647±0.013	7.83±0.502	3.49±0.211	0.446±0.01	0.92±0.195	0.838±0.024	21.513±0.722	0.093±0.004	0.474±0.012	0.376±0.045	0.114±0.01
ZBED6-/- Bama miniatures（n=6）	25.092±0.884	16.125±0.571	0.643±0.011	7.968±0.245	4.075±0.12	0.512±0.007	0.862±0.102	0.892±0.039	19.284±0.45	0.109±0.003	0.536±0.021	0.272±0.03	0.114±0.007
p-value	0.578	0.745	0.801	0.777	0.021*	0.0001***	0.763	0.29	0.015*	0.006**	0.029*	0.058	0.978

Growth performance and slaughter indexes between WT and ZBED6-/- female F4 pigs

Trait	body weight（kg）	carcass weight（kg）	slaughter rate（%）	left carcass weight（kg）	lean mass	lean meat rate（%）	pigskin weight (kg)	bone weight (kg)	back fat thickness(mm)	heart weight (kg)	liver weight (kg)	lung weight (kg)	kidney weight (kg)
WT Bama miniatures (n=5)	24.85	16.37	0.659	8	3.75	0.469	1.05	0.85	20.82	0.0828	0.448	0.334	0.1003
	27	18.5	0.685	9.25	3.95	0.427	1.55	0.838	24.09	0.09639	0.498	0.30051	0.141
	25.7	16.35	0.636	8.1	3.55	0.438	0.65	0.9	21.0325	0.1	0.471	0.325	0.129
	21.45	13.4	0.625	6.6	2.85	0.432	0.7	0.8	20.81	0.1	0.453	0.527	0.099
	22.8	14.35	0.629	7.2	3.35	0.465	0.65	0.8	20.81	0.084	0.5	0.391	0.1

Mean	24.36	15.794	0.647	7.83	3.49	0.446	0.92	0.838	21.513	0.093	0.474	0.376	0.114
SEM	1.115	0.993	0.013	0.502	0.211	0.01	0.195	0.024	0.722	0.004	0.012	0.045	0.01
	23.8	14.6	0.613	7.35	3.75	0.51	0.75	0.85	19.355	0.107	0.555	0.363	0.12
ZBED6-/-	28.2	17.65	0.626	8.45	4.15	0.491	1.3	0.9	18.4325	0.112	0.576	0.259	0.14
Bama	24.6	16.85	0.685	8.25	4.4	0.533	0.75	1	18.4075	0.118	0.566	0.186	0.1
miniatures	23.3	15.15	0.650	7.56	3.85	0.509	0.75	0.75	20.285	0.099	0.493	0.303	0.107
（n=6）	23.8	15.25	0.641	7.55	3.95	0.523	0.7	0.9	18.5275	0.107	0.566	0.313	0.095
	26.85	17.25	0.642	8.65	4.35	0.503	0.919	0.95	20.6975	0.108	0.459	0.205	0.123
Mean	25.092	16.125	0.643	7.968	4.075	0.512	0.862	0.892	19.284	0.109	0.536	0.272	0.114
SEM	0.884	0.571	0.011	0.245	0.12	0.007	0.102	0.039	0.45	0.003	0.021	0.03	0.007
p-value	0.578	0.745	0.801	0.777	0.021*	0.0001***	0.763	0.29	0.015*	0.006**	0.029*	0.058	0.978

Growth performance and slaughter indexes between WT and ZBED6-/- male F4 pigs

Trait	body weight（kg）	carcass weight（kg）	slaughter rate（%）	left carcass weight（kg）	lean mass	lean meat rate（%）	pigskin weight (kg)	bone weight (kg)	back fat thickness(mm)	heart weight (kg)	liver weight (kg)	lung weight (kg)	kidney weight (kg)
WT Bama miniatures (n=3)	21.733±0.195	13.15±0.061	0.605±0.003	6.467±0.02	3.383±0.147	0.523±0.022	0.867±0.041	1.05±0.071	13.624±0.263	0.1±0.008	0.6±0.048	0.402±0.054	0.164±0.004
ZBED6-/- Bama miniatures（n=3）	21.3±0.869	13.65±0.743	0.64±0.014	6.733±0.592	4±0.221	0.596±0.021	0.917±0.054	1.017±0.114	10.026±0.857	0.133±0.006	0.535±0.043	0.315±0.045	0.143±0.018
p-value	0.583	0.457	0.041*	0.611	0.047*	0.043*	0.417	0.776	0.016*	0.032*	0.369	0.284	0.321

Growth performance and slaughter indexes between WT and ZBED6-/- male F4 pigs

Trait	body weight（kg）	carcass weight（kg）	slaughter rate（%）	left carcass weight（kg）	lean mass	lean meat rate（%）	pigskin weight (kg)	bone weight (kg)	back fat thickness(mm)	heart weight (kg)	liver weight (kg)	lung weight (kg)	kidney weight (kg)
WT Bama	21.55	13.1	0.608	6.45	3.15	0.488	0.8	1.15	13.755	0.084	0.506	0.507	0.165
miniatures	21.6	13.1	0.606	6.45	3.55	0.55	0.9	1.05	13.1175	0.112	0.636	0.372	0.17
(n=3)	22.05	13.25	0.601	6.5	3.45	0.531	0.9	0.95	14	0.104	0.659	0.326	0.157
Mean	21.733	13.15	0.605	6.467	3.383	0.523	0.867	1.05	13.624	0.1	0.6	0.402	0.164
SEM	0.195	0.061	0.003	0.02	0.147	0.022	0.041	0.071	0.263	0.008	0.048	0.054	0.004
ZBED6-/-	20.8	13.65	0.65625	6.25	3.75	0.6	0.85	0.95	8.325	0.129	0.593	0.38	0.129
Bama	22.7	14.7	0.648	7.7	4.35	0.565	1	1.2	10.69	0.125	0.562	0.335	0.179
miniatures [illegible]	20.4	12.6	0.618	6.25	3.9	0.624	0.9	0.9	11.0625	0.144	0.451	0.229	0.121
Mean	21.3	13.65	0.64	6.733	4	0.596	0.917	1.017	10.026	0.133	0.535	0.315	0.143
SEM	0.869	0.743	0.014	0.592	0.221	0.021	0.054	0.114	0.857	0.006	0.043	0.045	0.018
p-value	0.583	0.457	0.041*	0.611	0.047*	0.043*	0.417	0.776	0.016*	0.032*	0.369	0.284	0.321
