# Supplementary material for: Porcine ZBED6 regulates growth of skeletal muscle and internal organs via multiple targets
Source: PLoS Genet. 2021 Oct 28;17(10):e1009862. doi: 10.1371/journal.pgen.1009862 (PMC8577783; doi:10.1371/journal.pgen.1009862)
Supplement: S4 Table — (PDF) [file pgen.1009862.s007.pdf]

The quality analysis and genome mapping analysis of transcriptome sequencing

Sample	Raw Reads	Clean Reads	Clean ratio	CleanQ30	mapped ratio
gastrocnemius muscle1	51324141	48827344	95.14	96.15;90.72	0.9406
gastrocnemius muscle2	54853833	52611224	95.91	96.09;90.44	0.9319
gastrocnemius muscle3	42609545	41157473	96.59	96.14;91.23	0.9405
gastrocnemius muscle4	51621850	49225625	95.36	96.40;91.46	0.9387
gastrocnemius muscle5	48534540	46469050	95.74	96.30;90.12	0.9388
gastrocnemius muscle6	39807691	37724044	94.77	96.34;90.82	0.9409
heart1	40241698	38971687	96.84	95.76;88.49	0.938
heart2	56977284	54157864	95.05	95.94;89.49	0.9269
heart3	44460101	42764905	96.19	96.21;91.73	0.9298
heart4	45403659	43618327	96.07	96.20;91.71	0.9297
heart5	49894338	45301686	90.8	95.89;88.46	0.9262
heart6	54203615	51972678	95.88	96.19;90.55	0.931
liver1	40991220	39086945	95.35	94.77;87.19	0.9232
liver2	42309385	40168212	94.94	94.49;86.92	0.9083
liver3	42913766	40018061	93.25	95.64;89.11	0.9208
liver4	51520282	48817500	94.75	96.34;90.94	0.9398
liver5	55517131	52825050	95.15	95.78;87.87	0.9205
liver6	53019323	49965249	94.24	95.92;90.10	0.9351
longissimus dorsi1	41296640	39852331	96.5	96.14;90.24	0.9433
longissimus dorsi2	47292326	45779690	96.8	96.07;89.54	0.9378
longissimus dorsi3	51741894	49156059	95	96.26;91.61	0.9462
longissimus dorsi4	47570197	45443444	95.53	96.36;89.76	0.9438
longissimus dorsi5	48685438	46505728	95.52	95.92;90.97	0.9402
longissimus dorsi6	55057017	52930196	96.14	96.13;90.03	0.9434
