# Supplementary material for: Porcine ZBED6 regulates growth of skeletal muscle and internal organs via multiple targets
Source: PLoS Genet. 2021 Oct 28;17(10):e1009862. doi: 10.1371/journal.pgen.1009862 (PMC8577783; doi:10.1371/journal.pgen.1009862)
Supplement: S5 Table — (PDF) [file pgen.1009862.s008.pdf]

The DEGs of heart between WT and ZBED6-/- pigs

Heart_WT_mean	Heart_ZBED6-/- _mean	GeneID	gene name	log2FoldChang	pvalue	padj
37.05491703	79.36106635	;SSCG00000035	IGF2	1.098766416	5.67846E-05	0.015126824
11.00096069	28.52695677	;SSCG00000001	CDKN1A	1.374696333	0.000331068	0.046493368
71.05161451	11.77631009	;SSCG00000017	SERPINF1	-1.727369946	4.95E-10	0.000000466
0.07890952	0.557339302	;SSCG00000034	RTN4R	1.657307373	1.17E-08	0.00000784
1.482235931	4.288089028	;SSCG00000023	CPM	1.126801659	0.0000321	0.006657327
35768.30686	467.4429019	;SSCG00000035	SSCG00000035	-4.949186722	2.36E-100	3.33E-96
9.190515989	53.39610562	;SSCG00000012	MX1	1.961013764	5.3E-15	1.5E-11
3.229790196	12.20385975	;SSCG00000017	DHX58	1.660225428	7.14E-15	1.68E-11
4.72470016	17.45078255	;SSCG00000012	MX2	1.605531503	6.4E-13	1.13E-09
0.600718864	4.30962959	;SSCG00000009	PLAC8	1.928357412	1.55E-12	2.43E-09
5.337169703	19.12498623	;SSCG00000008	CMPK2	1.533482531	3.71E-11	4.03E-08
67.1192326	433.3104974	;SSCG00000035	ISG12(A)	1.715387491	1.03E-09	0.000000909
11.08793427	37.27341049	;SSCG0000000(	USP18	1.383305969	1.09E-08	0.00000767
3.713817816	12.70258379	;SSCG00000012	FAM155B	1.342417379	0.000000582	0.000234396

0.10839656	0.661007019	SSCG00000029	AOX1	1.413548847	0.00000154	0.00054263
46.47770754	2363.161301	SSCG00000018	ATP8	1.328168672	0.00000189	0.000648923
4.894821924	0.039651234	SSCG0000003	SSCG00000031	-1.19684191	0.0000125	0.003158439
0.085701664	8.419752609	SSCG00000039	SLC9A3	4.034644397	1.12E-47	7.93E-44
1505.01248	91.75741601	SSCG00000016	GCK	-2.62744428	1.29E-21	6.08E-18
6.714947343	0.533045923	SSCG00000039	CES1	-2.39466794	2.46E-18	8.67E-15
6.399548936	1.138589724	SSCG00000016	AASS	-1.849211383	6.77E-14	1.36E-10
15.52579425	3.103965455	SSCG00000027	DHCR24	-1.750902458	2.06E-12	2.91E-09
3.809639446	15.55925302	SSCG00000008	RSAD2	1.654485905	3.53E-12	4.53E-09
0.50785819	2.687388084	SSCG00000018	SLC47A2	1.791782984	6.12E-12	7.2E-09
1.339023049	0.236108391	SSCG00000025	CHMP4C	-1.707618278	1.92E-10	0.000000193
81.26015447	29.31960783	SSCG0000000	LOC100127131	-1.221130562	1.36E-09	0.00000113
20.13026573	6.799893394	SSCG00000015	NIBAN1	-1.264216395	2.37E-09	0.00000186
78.70952888	19.90264556	SSCG00000002	FBLN5	-1.477181092	7.21E-09	0.00000535

5.274542824	18.91610771	SSCG00000016	PARP12	1.42332111	1.37E-08	0.00000877
1.462822131	0.34570786	SSCG00000011	PEX5L	-1.493394953	1.69E-08	0.0000104
15.95698851	3.288412794	SSCG00000017	SSCG00000017	-1.505699324	0.000000046	0.000027
0.024698425	0.525096702	SSCG00000002	CYP46A1	1.575973499	6.97E-08	0.0000382
6.36557064	15.23566784	SSCG00000003	IFI44	1.108843037	7.05E-08	0.0000382
4.160282474	11.75189671	SSCG00000033	CD163	1.242972547	7.87E-08	0.0000391
0.953696353	6.020672218	SSCG00000038	R-SSC-198933	1.559754495	8.03E-08	0.0000391
3.162909665	0.986411269	SSCG00000015	GRAMD1B	-1.270376991	7.86E-08	0.0000391
36.84634764	13.28023992	SSCG00000032	DAP	-1.194091478	0.000000142	0.0000668
2.985984583	8.095250859	SSCG00000015	IFIH1	1.190883208	0.000000231	0.000105341
6578.917964	18027.24992	SSCG00000018	ND1	1.222888512	0.000000266	0.000117045
4.3185539	0.901797379	SSCG00000006	AARD	-1.441812655	0.0000003	0.000128264
1.067305042	4.231156443	SSCG00000016	SKAP2	1.385748964	0.000000313	0.000129751
1.131935433	0.243462495	SSCG00000006	PLAG1	-1.40262745	0.000000634	0.00024854

0.496180656	2.786377025	SSSCG0000004(	CBS	1.433530952	0.00000104	0.00039713
6.074612143	17.37711474	SSSCG00000008	EIF2AK2	1.185131909	0.00000135	0.000499926
0.135222021	1.145098245	SSSCG00000009	OASL	1.422952989	0.00000143	0.000518499
18.46339111	5.285092244	SSSCG0000000(	GTSE1	-1.247529128	0.00000219	0.00073497
27.20089733	9.782499561	SSSCG00000036	LOC110258338	-1.126531136	0.00000271	0.000869163
6.748553892	15.88314913	SSSCG00000001	PML	1.068213577	0.00000268	0.000869163
1.158584194	2.996753012	SSSCG00000031	ACKR2	1.143732309	0.00000287	0.000900481
1.844847414	6.72736547	SSSCG00000038	SBK1	1.28492088	0.0000033	0.001013354
3.217306327	7.21974009	SSSCG00000034	RTP4	1.003842166	0.00000503	0.001509566
3.758612012	0.968761343	SSSCG00000025	GRIK1	-1.267541754	0.00000628	0.001845067
60.49732886	138.2241871	SSSCG00000002	SSCG00000002	1.033486657	0.00000654	0.001884212
3.476762172	12.79619584	SSSCG00000037	EPSTI1	1.255683535	0.00000765	0.002158146
6.992056639	19.77545729	SSSCG00000035	CCL24	1.144768133	0.00000936	0.002558456
5.826936759	2.363937265	SSSCG00000017	FBF1	-1.004547106	0.00000978	0.002601838

0.250791067	2.029180803	SSSCG00000037	SSSCG00000037	1.28362324	0.0000121	0.003114961
21.39380182	8.041667203	SSSCG00000006	TSPAN2	-1.088889092	0.0000121	0.003114961
4.441428161	16.62712476	SSSCG00000015	IL10RA	1.219612322	0.0000184	0.004336839
11.51529795	3.902968302	SSSCG00000016	ADAMTS6	-1.118169005	0.0000191	0.004406665
0.134582915	0.982456198	SSSCG00000028	SSSCG00000028	1.233589984	0.0000267	0.005794332
11.96067548	32.22081866	SSSCG00000037	R-SSC-202733	1.086857538	0.0000285	0.006098942
1.270781002	4.401833999	SSSCG00000035	ADGRB1	1.1929419	0.0000294	0.006192294
24.01029761	9.146965026	SSSCG00000015	NMNAT2	-1.000342236	0.0000543	0.010940917
1.286177749	0.19405849	SSSCG00000040	F8A1	-1.173288982	0.0000668	0.013266969
0.229936192	1.368261939	SSSCG00000024	SSSCG00000024	1.152075174	0.000091	0.017836573
0.048852767	0.439233404	SSSCG00000032	CPZ	1.117119535	0.0000995	0.019234024
0.68234378	1.954445425	SSSCG00000013	MS4A7	1.06452815	0.000122092	0.023275043
0.023223634	0.196041329	SSSCG00000016	LOC100522404	1.090377205	0.00014511	0.026585334
0.045622957	0.221133614	SSSCG00000002	CDH15	1.115793139	0.000156396	0.02828565

0.752357961	0.040227988	SSSCG00000005	PLPPR1	-1.028784069	0.000162079	0.028580638
0.431032062	0.102411814	SSSCG00000004	MNS1	-1.10471665	0.000164477	0.028645344
2.165531796	9.853014949	SSSCG00000023	SSSCG00000023	1.1085868	0.000172236	0.029273831
0.186083688	0.53868519	SSSCG00000003	NAPSA	1.050192662	0.000199921	0.032758446
0.112994135	0.49944252	SSSCG00000015	ST14	1.093229667	0.00021105	0.033832686
0.188259646	0.72601385	SSSCG00000002	ADGRG3	1.075274358	0.000247357	0.037928934
1.90973871	5.406487739	SSSCG00000028	TMEM52	1.034623341	0.000246515	0.037928934
0.514238965	2.607110973	SSSCG00000013	BDNF	1.079811547	0.000251488	0.038147734
0.628928346	0.060688834	SSSCG00000003	R-SSC-6798695	-1.027375717	0.000266087	0.039512453
1.312808943	3.406719909	SSSCG00000017	MEOX1	1.004802841	0.00029442	0.042244615
0.781514065	0.105177144	SSSCG00000038	FYB2	-1.032998116	0.000343542	0.046621482
0.639002761	0.153836452	SSSCG00000010	TLL2	-1.053560342	0.000337628	0.046621482
2.613898179	0.812016886	SSSCG00000015	THSD7A	-1.019182272	0.000368391	0.048569097
1.083507645	0.311757756	SSSCG00000007	CNBD2	-1.029339248	0.000372349	0.048636309

Note : ZBED6 targets are in red type.
