# Supplementary material for: Porcine ZBED6 regulates growth of skeletal muscle and internal organs via multiple targets
Source: PLoS Genet. 2021 Oct 28;17(10):e1009862. doi: 10.1371/journal.pgen.1009862 (PMC8577783; doi:10.1371/journal.pgen.1009862)
Supplement: S6 Table — (PDF) [file pgen.1009862.s009.pdf]

The DEGs of gastrocnemius muscle between WT and ZBED6-/- pigs

GM_WT_mean	GM_ZBED6-/- _mean	GeneID	gene name	log2FoldChange	pvalue	padj
7.096858639	44.60156787	ENSSSCG00000040575	ISG15	1.924675022	7.10E-11	1.90E-07
0.03929823	0.340534729	ENSSSCG00000011416	DOCK3	1.766977875	5.41E-08	8.25E-05
35.92565709	72.30328329	ENSSSCG00000035293	IGF2	1.009046617	3.98E-06	0.002366401
13.03388759	26.14499287	ENSSSCG00000001565	CDKN1A	1.004267219	0.000100951	0.030978617
7.053369958	17.92610985	ENSSSCG00000031888	DDIT4	1.219848019	1.41E-06	0.001192671
10.51281449	0.406020047	ENSSSCG00000031101	METTL21C	-2.546930916	9.88E-16	5.29E-12
0.736426853	7.901435658	ENSSSCG00000001550	ARMC12	2.098800743	4.13E-11	1.33E-07
34.33707024	7.033609321	ENSSSCG00000000194	GTSE1	-1.659648883	1.32E-10	3.03E-07
0.663110808	2.207992918	ENSSSCG00000030548	HERC5	1.520961211	1.83E-09	3.68E-06
0.041407076	0.420056993	ENSSSCG00000034914	CD163L1	1.767262027	5.65E-08	8.25E-05
2.449091105	5.45208365	ENSSSCG00000016502	PARP12	1.153302737	1.94E-07	0.000259295
14.16284607	31.89824872	ENSSSCG00000009881	OAS2	1.138171599	7.24E-07	0.000811739
2.652355306	7.722775002	ENSSSCG00000008648	RSAD2	1.304370231	7.99E-07	0.000811739

0.979812289	2.739807852	ENSSSCG00000027847	CSKMT	1.325359107	9.98E-07	0.000896457
1.893966885	0.342673674	ENSSSCG00000040321	F8A1	-1.494835622	1.77E-06	0.001409205
0.662026172	2.090255845	ENSSSCG00000009720	DDX60	1.370710549	1.93E-06	0.001409205
0.705317473	0.13359296	ENSSSCG00000039332	SEC16B	-1.4530028	2.03E-06	0.001419623
2557.630902	8086.472382	ENSSSCG00000018065	ND1	1.31426755	2.99E-06	0.001922867
4.392482776	1.118192796	ENSSSCG00000027607	IER3	-1.322848526	3.54E-06	0.002189095
1.793555289	9.674419141	ENSSSCG00000032451	ENSSSCG00000032451	1.480785045	4.20E-06	0.002407178
3.873607341	8.152987089	ENSSSCG00000030408	DDX58	1.037894393	6.36E-06	0.003403694
23.21851764	63.52546601	ENSSSCG00000016057	STAT1	1.23392189	1.05E-05	0.005418599
14.69885606	59.73956355	ENSSSCG00000013400	MICAL2	1.376572395	1.11E-05	0.005474671
4.719403176	0.879300918	ENSSSCG00000040769	LOC110257712	-1.380943601	1.12E-05	0.005474671
4.844659283	13.86537851	ENSSSCG00000016200	PRKAG3	1.211236295	1.97E-05	0.009058926
8.629997192	23.937901	ENSSSCG00000010452	IFIT1	1.167220609	4.32E-05	0.017351323
0.841561164	4.737990524	ENSSSCG00000002279	GPX2	1.321803434	4.88E-05	0.019103821

2435.845296	6153.413093	ENSSSCG00000018069	ND2	1.104603475	5.42E-05	0.020430895
3.243725187	0.637451332	ENSSSCG00000009364	FREM2	-1.270236218	5.47E-05	0.020430895
0.084640841	0.292707029	ENSSSCG00000035805	DLK1	1.243577142	6.74E-05	0.023545566
0.615577648	0.080926691	ENSSSCG00000007261	BPIFB1	-1.295993283	6.95E-05	0.023767541
0.911140235	2.254643868	ENSSSCG00000037520	ZNF275	1.137432709	7.55E-05	0.025268853
6.204664623	16.50167128	ENSSSCG00000021712	HERC6	1.141637863	7.95E-05	0.026074551
0.953854026	3.356432995	ENSSSCG00000037572	EPSTI1	1.25028767	8.43E-05	0.026560617
0.588114079	0.057160124	ENSSSCG00000002811	CNGB1	-1.233669861	0.000130878	0.03893609
30.16164063	87.70969392	ENSSSCG00000037775	R-SSC-202733	1.128580422	0.000136696	0.039927724
2.395955201	0.415753414	ENSSSCG00000037106	ECRG4	-1.229335132	0.000140306	0.040250405
13537.11502	223.4308952	ENSSSCG00000035520	ENSSSCG00000035520	-3.56345424	1.38E-30	2.21E-26
21.94193623	1194.98784	ENSSSCG00000018080	ATP8	3.184236799	2.12E-23	1.70E-19
4.771926749	14.27884964	ENSSSCG00000000774	USP18	1.515934362	2.31E-11	9.26E-08
0.165657652	1.319184539	ENSSSCG00000009240	PLAC8	1.757761547	4.82E-08	8.25E-05

8.449141161	22.45993617	ENSSSCG00000035297	ISG12(A)	1.261334191	3.31E-07	0.000409009
6.593783715	19.58398629	ENSSSCG00000012077	MX1	1.332862879	8.08E-07	0.000811739
0.041106511	0.252392865	ENSSSCG00000029606	AOX1	1.582382868	1.00E-06	0.000896457
2.798117862	6.559275725	ENSSSCG00000008647	CMPK2	1.1653153	1.88E-06	0.001409205
3.305084538	9.009184516	ENSSSCG00000012076	MX2	1.246188906	2.41E-06	0.001611519
0.994699365	4.532191623	ENSSSCG00000012386	FAM155B	1.465811305	5.42E-06	0.003001639
5.57992168	0.194062436	ENSSSCG00000031306	ENSSSCG00000031306	-1.323941996	2.80E-05	0.012166283
1.498181551	3.315182218	ENSSSCG00000017416	DHX58	1.04388693	6.34E-05	0.022651021

Note : ZBED6 targets are in red type.
