# Supplementary material for: Porcine ZBED6 regulates growth of skeletal muscle and internal organs via multiple targets
Source: PLoS Genet. 2021 Oct 28;17(10):e1009862. doi: 10.1371/journal.pgen.1009862 (PMC8577783; doi:10.1371/journal.pgen.1009862)
Supplement: S7 Table — (PDF) [file pgen.1009862.s010.pdf]

The DEGs of longissimus dorsi between WT and ZBED6-/- pigs

LD_WT_mean	LD_ZBED6-/- _mean	GeneID	gene name	log2FoldChange	pvalue	padj
26.14210446	81.05518197	ENSSSCG000000352[illegible]	IGF2	1.474023158	6.16E-06	0.004210493
90.46999529	210.2730082	ENSSSCG00000001565	CDKN1A	1.216752368	0.00013583	0.04067824
0.012641522	0.176637477	ENSSSCG00000011416	DOCK3	2.591252255	3.65E-11	1.37E-07
0.161636439	1.53328755	ENSSSCG00000034656	RTN4R	2.443502036	5.73E-11	1.37E-07
6.294079801	31.08448875	ENSSSCG00000040575	ISG15	1.780283506	2.64E-06	0.002228436
0.075314636	0.274847346	ENSSSCG00000003214	KCNC3	1.522924404	4.24E-05	0.019043475
0.071209154	0.247419476	ENSSSCG00000032360	PANX1	1.411332558	0.000151868	0.04345611
11.68790708	325.0761344	ENSSSCG00000018080	ATP8	3.722749901	2.18E-25	3.13E-21
7075.495598	132.0171797	ENSSSCG00000035520	ENSSSCG00000035520	-3.88516375	1.50E-23	1.08E-19
0.007662875	0.218527435	ENSSSCG00000029606	AOX1	2.142487737	4.37E-07	0.000628159
3.016111482	10.76944103	ENSSSCG00000012077	MX1	1.591519956	1.94E-06	0.001985394
0.128137684	0.801304261	ENSSSCG00000009240	PLAC8	1.790474978	5.46E-06	0.003919579
1.018278907	4.469103891	ENSSSCG00000012386	FAM155B	1.631185163	3.06E-05	0.015689119
1.37693384	3.648749984	ENSSSCG00000008647	CMPK2	1.298056002	3.30E-05	0.016319629

2.689547288	7.463696834	ENSSSCG00000000774	USP18	1.333648391	5.61E-05	0.022378892
1.486574211	3.74936379	ENSSSCG00000012076	MX2	1.218561087	6.58E-05	0.025529493
3.341184999	0.157572009	ENSSSCG00000031306	ENSSSCG00000031306	-1.660809617	8.28E-05	0.029707634
0.731305439	1.998796163	ENSSSCG00000017416	DHX58	1.297973539	0.00012235	0.039925123
5.809995805	17.27268583	ENSSSCG00000035297	ISG12(A)	1.321232405	0.00014484	0.042441004
1.499014497	23.90079841	ENSSSCG00000036983	ENSSSCG00000036983	2.614438173	4.14E-11	1.37E-07
0.528492027	4.51361065	ENSSSCG00000037155	ENSSSCG00000037155	2.393889875	5.64E-11	1.37E-07
0.494301654	0.02224188	ENSSSCG00000002811	CNGB1	-2.521422375	1.26E-09	2.58E-06
0.081291602	0.589723208	ENSSSCG00000017187	FOXJ1	2.103491882	3.35E-08	6.02E-05
0.470158961	5.245951208	ENSSSCG00000032451	ENSSSCG00000032451	2.243441365	4.09E-08	6.52E-05
0.666818459	0.139079916	ENSSSCG00000026978	ROS1	-1.68960274	6.03E-07	0.000786564
0.143306849	1.19251002	ENSSSCG00000038048	NECAB2	2.00349297	1.00E-06	0.001196715
4.62037352	1.089199638	ENSSSCG00000008535	CLIP4	-1.63355907	1.52E-06	0.001673699
0.54486732	0.006557957	ENSSSCG00000037929	H2BC11	-1.990861429	2.54E-06	0.002228436

0.323808204	4.105307855	ENSSSCG00000037009	ENSSSCG00000037009	1.978783327	2.58E-06	0.002228436
0.146160531	0.708098176	ENSSSCG00000017372	MPP3	1.757715171	3.31E-06	0.002643841
0.491452696	0.017819153	ENSSSCG00000038184	ACBD7	-1.955518711	4.04E-06	0.003050336
0.105875303	0.45848779	ENSSSCG00000033214	GNMT	1.6528203	6.98E-06	0.004557966
0.672038907	3.666693535	ENSSSCG00000001550	ARMC12	1.752359053	1.17E-05	0.007327043
2.113254426	6.374405501	ENSSSCG00000017194	CDK3	1.399073254	1.72E-05	0.010290622
0.153239516	0.737660019	ENSSSCG00000033413	NRTN	1.653901754	1.83E-05	0.010506348
0.102040503	1.037400285	ENSSSCG00000007760	PRSS36	1.78224431	2.41E-05	0.013288283
0.489150316	3.760116646	ENSSSCG00000038719	ENSSSCG00000038719	1.72700272	2.87E-05	0.015258683
0.352671675	0.014888694	ENSSSCG00000038591	ENSSSCG00000038591	-1.74724454	3.66E-05	0.017499503
2.293368665	0.418140702	ENSSSCG00000015375	ITGB8	-1.623937805	4.17E-05	0.019043475
0.457423478	2.302863638	ENSSSCG00000002009	PCK2	1.617015531	4.57E-05	0.019894143
9.768924492	61.28406661	ENSSSCG00000030300	MT-2B	1.664519247	4.82E-05	0.020356647
2.182232897	0.737038915	ENSSSCG00000032450	LYRM9	-1.262079061	4.98E-05	0.020418784

3.596001536	9.27509094	ENSSSCG00000014830	COA4	1.222964117	6.79E-05	0.025640842
4.472311716	0.765521328	ENSSSCG00000031101	METTL21C	-1.629515717	7.20E-05	0.026501365
1.041480631	4.53460857	ENSSSCG00000022738	LOC100519130	1.521588323	9.50E-05	0.033263362
0.214132079	0	ENSSSCG00000014984	MMP27	-1.597194832	0.000102133	0.034914941
7.394807565	17.10808792	ENSSSCG00000009881	OAS2	1.144008019	0.000112714	0.037635896
2.001431387	0.623715022	ENSSSCG00000003699	GREB1L	-1.323998358	0.000128465	0.040203571
3.49592697	8.168627673	ENSSSCG00000023379	UBE2L6	1.177329467	0.000129661	0.040203571
0.321743211	2.448177442	ENSSSCG00000003753	PDZK1IP1	1.605920754	0.000131604	0.040203571
0.37276337	0.945477456	ENSSSCG00000030548	HERC5	1.216578904	0.000136256	0.040757516
0.959249362	0.304259847	ENSSSCG00000036201	NPR3	-1.314137581	0.000154357	0.04345611
0.075414713	0.819831505	ENSSSCG00000040651	ENSSSCG00000040651	1.589264842	0.000179526	0.049569935

Note : ZBED6 targets are in red type.
