# Supplementary material for: Porcine ZBED6 regulates growth of skeletal muscle and internal organs via multiple targets
Source: PLoS Genet. 2021 Oct 28;17(10):e1009862. doi: 10.1371/journal.pgen.1009862 (PMC8577783; doi:10.1371/journal.pgen.1009862)
Supplement: S8 Table — (PDF) [file pgen.1009862.s011.pdf]

The DEGs of liver between WT and ZBED6-/- pigs

Liver_WT_mean	Liver_ZBED6-/-_mean	GeneID	gene name	log2FoldChange	pvalue	padj
0.073397441	0.967704856	ENSSSCG00000031957	RIMS2	1.965642856	0.000000017	0.0000228
0.349316657	1.450832277	ENSSSCG00000034656	RTN4R	1.416503084	0.000013	0.005457824
31.17486403	105.6855506	ENSSSCG00000014871	TSKU	1.761322971	0.0000808	0.02653143
16.25585763	46.26978081	ENSSSCG00000010447	ACTA2	1.165588462	0.000175477	0.049720643
25.98541418	62.55097479	ENSSSCG00000004578	ANXA2	1.130317919	0.0000151	0.006182374
136.3939724	9.494782021	ENSSSCG00000037268	APCS	-2.900294387	1.21E-23	8.93E-20
44.69642582	6.051425929	ENSSSCG00000038777	C7orf50	-2.0928618	9.81E-12	2.41E-08
0.643680094	2.113130213	ENSSSCG00000035887	C8orf34	1.414857736	0.00000098	0.000601561
2.744764084	9.591273841	ENSSSCG00000008647	CMPK2	1.527065372	6.81E-08	0.0000772
0.030277073	0.349250321	ENSSSCG00000012164	CNKSR2	2.169203137	1.77E-10	0.000000261
4.591915137	1.038412244	ENSSSCG00000016557	CPA1	-1.57583234	0.000000112	0.000117766
3.867539003	9.984822189	ENSSSCG00000017416	DHX58	1.191102048	0.0000119	0.005139591
2.398095339	5.725731031	ENSSSCG00000003417	DISP3	1.119539423	0.0000402	0.014439067

1.263118699	0.066767683	ENSSSCG00000016258	DNER	-2.2761573	3.86E-11	6.58E-08
1.92709019	0.292631494	ENSSSCG00000000400	MIP	-1.588767395	0.00000279	0.001467676
1.50692538	0.10587455	ENSSSCG00000003278	R-SSC-6798695	-2.25079354	2.44E-11	5.14E-08
1.346737292	10.75608193	ENSSSCG00000009240	PLAC8	1.786636883	0.000000196	0.000180065
0.020722786	0.169380797	ENSSSCG00000014894	TENM4	1.780249931	0.00000023	0.000188329
16.52331565	2.773378141	ENSSSCG00000026167	ENSSSCG00000026167	-1.71212769	0.000000146	0.00014309
9.085614838	23.10516461	ENSSSCG00000030408	DDX58	1.216061089	0.00000379	0.00192536
10.51674599	0.281162351	ENSSSCG00000031306	ENSSSCG00000031306	-1.913426854	4.18E-08	0.0000513
1.181637654	0	ENSSSCG00000034586	ENSSSCG00000034586	-1.783226501	0.000000208	0.000180065
2.384178123	0.223382268	ENSSSCG00000040321	F8A1	-2.248476344	2.95E-12	8.69E-09
1.062198037	2.971003585	ENSSSCG00000030548	HERC5	1.276027119	0.00000523	0.002486625
7.41858352	20.99854617	ENSSSCG00000003763	IFI44	1.270430212	0.0000052	0.002486625
12.77925318	34.14846264	ENSSSCG00000010452	IFIT1	1.267714287	0.000000456	0.000335935
4.482733232	9.830440128	ENSSSCG00000010454	IFIT5	1.027269652	0.00005	0.017540101

0.0037953	0.326897706	ENSSSCG00000013341	LUZP2	1.251151945	0.000131177	0.037897335
0.790931074	2.647150035	ENSSSCG00000015716	MARCO	1.342003559	0.0000102	0.004564308
6.238735698	0.95167463	ENSSSCG00000035366	MFHAS1	-2.253330136	2.15E-20	1.06E-16
31.4667295	12.29577797	ENSSSCG00000003898	MKNK1	-1.09664873	0.00000253	0.001434688
1.200780551	2.968736125	ENSSSCG00000017605	MMD	1.125192809	0.000076	0.025445945
14.16123173	68.74482492	ENSSSCG00000012077	MX1	2.03177605	1.52E-16	5.62E-13
1.859563072	7.313731371	ENSSSCG00000012076	MX2	1.509245411	0.00000108	0.000635242
7026.87269	266.3300028	ENSSSCG00000035520	ENSSSCG00000035520	-2.301460956	4.02E-11	6.58E-08
1.630837211	0.021826178	ENSSSCG00000037464	ENSSSCG00000037464	-1.698410782	0.000000757	0.00050719
0.112076142	1.551977792	ENSSSCG00000028816	ENSSSCG00000028816	1.474346067	0.0000213	0.008247182
1248.709005	5133.562832	ENSSSCG00000018065	ND1	1.398891809	0.0000276	0.01017781
1258.037676	4879.299381	ENSSSCG00000018069	ND2	1.383791486	0.0000248	0.009386428
25.00458552	9.099828656	ENSSSCG00000003565	NR0B2	-1.095391562	0.0000891	0.027923143

0.035722405	3.18545705	ENSSSCG00000006791	OVGP1	4.27256611	2.07E-42	3.04E-38
7.370153583	16.40941419	ENSSSCG00000003551	PAQR7	1.020731507	0.000120451	0.035494444
6.075685722	0.999202871	ENSSSCG00000004452	PRSS35	-1.355271299	0.000101136	0.030411029
0.888305825	3.042379393	ENSSSCG00000008648	RSAD2	1.346314594	0.0000156	0.006231518
10.99187757	2.653641308	ENSSSCG00000027806	SAMHD1	-1.402193046	0.00000784	0.003609613
2.818745744	6.341835275	ENSSSCG00000028923	SCNN1B	1.052585867	0.0000791	0.025893665
0.199338545	0.749980378	ENSSSCG00000038946	SLC17A8	1.491877152	0.000000823	0.00052703
0.21307346	0.028679166	ENSSSCG00000040453	SYN3	-1.37822935	0.0000814	0.026070679
1.768885024	4.929918287	ENSSSCG00000032431	TMEM52B	1.193822183	0.0000693	0.023749801
6.547616129	17.26469807	ENSSSCG00000014670	TRIM22	1.267998432	0.000000406	0.000314469
0.812101309	0.073164834	ENSSSCG00000022945	UCHL1	-1.643065478	0.00000263	0.001434688
1.562953249	5.824538811	ENSSSCG00000007508	ZBP1	1.504299512	0.000000639	0.000448255

Note : ZBED6 targets are in red
