# Supplementary material for: Porcine ZBED6 regulates growth of skeletal muscle and internal organs via multiple targets
Source: PLoS Genet. 2021 Oct 28;17(10):e1009862. doi: 10.1371/journal.pgen.1009862 (PMC8577783; doi:10.1371/journal.pgen.1009862)
Supplement: S9 Table — (PDF) [file pgen.1009862.s012.pdf]

Test	Primer name	Forward (5′-3′)	Reverse (5′-3′)	Annealing temperature/°C	Product size/bp
PCR	IGF2-1	CTCCGGGGACTGTTGAAGT	GAAGGGAGGAAGCCGAGAG	56	291
	ZBED6-1	GGTGGCAGAAGGAGTGGATAAA	CTCCTCCCCACTAGTAACTCCA	56	451
qPCR	GAPDH	AGAGATAAGGAGTGTGTCT	CCAACGAAGAGGAAGAAT	60	168
	18S	GACGTGACTGCTCGGTGC	AACTCGACCGAGGGCACAAG	60	144
	β-actin	TCTGGCACCACACCTTCTA	AAGGTCTCGAACATGATCTG	60	127
	IGF2-2	GTGGCATCGTGGAAGAGTGC	CCAGGTGTCATAGCGGAAGAA	60	167
	ZBED6-2	ATTGCCTGCCAAAAAGAAAA	CAACATGAAAGAAGTGCCAC	60	246
	ISG12(A)	ATTCAAGGCTGCCCTAACCG	GAAGCCTTGGCTCCTAGTGAA	60	126
	STAT1	TACAGGCACTCTGCTGTCTCC	GAATGTCACCGAACTTACCCA	60	80
	MX1	GAGGCAGCGGAATTGTGAC	TCCCGGTAACTGACTTTGCC	60	164
	MX2	CCTTGAGGTACAGCCCGTTC	CACCTGGTGATGATTCCGCT	60	199
	RTN4R	TACAACGAGCCCAAGGTGAC	GCACGTATGTGATGCGGTTC	60	187
	ATP1A3	AGCTGAACTTTCCCACGGAG	AAATGATGCCCACGCCTTTG	60	180
ChIP-PCR	IGF2-3	ACAAGCTGTGTCCAGTGTACT	AAAGGCAACTGCTGAACCCT	59	196
	CDKN1A	CCAGAGAGACCCCTTTTTCAGG	AGTCCAATCCCATCAGCAAGC	59	186
	TSKU	ATCAAGCCCTGGTTTTGAGG	CGAATCTGCCTCCTCATGAA	59	164
	GNAZ	CGAGAGCAAGGGTGAGATCA	CGCTCTAGGTCGTTCAGGTA	59	144
